# Analysis of the Relationship Between *NF-κB1* and Cytokine Gene Expression in Hematological Malignancy: Leveraging Explained Artificial Intelligence and Machine Learning for Small Dataset Insights

**DOI:** 10.7150/ijms.109493

**Published:** 2025-04-13

**Authors:** Jae-Seung Jeong, Hyunsu Ju, Chi-Hyun Cho

**Affiliations:** 1 Division of Artificial Intelligence Convergence Engineering, Sahmyook University, 01795, Republic of Korea.; 2 Post-Silicon Semiconductor Institute, Korea Institute of Science and Technology, 02792, Republic of Korea.; 3 Department of Laboratory Medicine, College of Medicine, Korea University Ansan Hospital, 15355, Republic of Korea.

**Keywords:** NF-κB / Hematological Malignancy / Machine Learning Classifiers / Explainable Artificial Intelligence / Small Data Adaptation

## Abstract

This study measures expression of *nuclear factor kappa B* (*NF-κB*)*1* and related cytokine genes in bone marrow mononuclear cells in patients with hematological malignancies, analyzing the relationship between them with an integrated framework of statistical analyses, machine learning (ML), and explainable artificial intelligence (XAI). While traditional dimensionality reduction techniques—such as principal component analysis, linear discriminant analysis, and t-distributed stochastic neighbor embedding—showed limited differentiation embedding, ML classifiers (k-Nearest Neighbors, Naïve Bayes Classifier, Random Forest, and XGBoost) successfully identified critical patterns. Notably, normalized caspase-1 counts consistently emerged as the most influential feature associated with NF-κB1 activity across disease groups, as highlighted by SHapley Additive exPlanations analyses. Systematic evaluation of ML performance on small datasets revealed that a minimum sample size of 15-24 is necessary for reliable classification outcomes, particularly in cohorts of acute myeloid leukemia and myelodysplastic syndrome. These findings underscore the pivotal role of caspase-1 to the NF-κB1 gene expression in hematologic malignancy diseases. Furthermore, this study demonstrates the feasibility of leveraging ML and XAI to derive meaningful insights from limited data, offering a robust strategy for biomarker discovery and precision medicine in rare hematological malignancies.

## Introduction

Hematological malignancies involve complex genetic interactions, with nuclear factor kappa B (NF-κB) playing a key role in regulating immune responses and inflammation within the bone marrow 1-3. NF-κB has been identified as a critical oncogenic driver in various hematological cancers, remarkably influencing disease progression and patient outcomes 4-6. Despite its pivotal role, the detailed mechanisms linking *NF-κB* with other cytokine genes remain inadequately explored, leaving crucial gaps in understanding its functional interactions and implications for disease management.

The NF-kB transcription factor family comprises five members, including NF-kB1 (the mature p50 and its precursor, p105), NF-kB2 (the mature p52 and its precursor, p100), RelA (p65), RelB, and c-Rel 7. These assemble into various homo- and heterodimeric combinations to regulate the expression of a large number (>500) of target genes, including those of cytokines, chemokines, growth factors, apoptosis regulators, cell surface receptors, and other transcription factors 7. P50 or p52 homodimer acts as a transcriptional repressor, while the dimers containing RelA (p65), RelB and c-Rel act as transcriptional activators and cause different reactions depending on the combination of the dimers 8, 9.

In the canonical pathway, for transcriptional activation, p50 is required to heterodimerize with RelA, which contains a transactivation domain 7, 8. Transcriptional activation via the canonical pathway is characterized by instant and reversible expression of target genes encoding inflammatory cytokines, such as IL-6, and immune response genes 7. In line with the relationship of p50 to such many genes, recently haploinsufficiency of the NF-kB1 subunit p50 was reported to be associated with a common variable immunodeficiency (CVID) phenotype 7.

While p50 is encoded by *NF-κB1* gene​,​​ *NF-κB1* gene interacts with numerous genes, including *caspase-1*, myeloid differentiation primary response 88 (*MyD88*), *interferon beta 1*, and *tumor necrosis factor*, which regulate key processes such as inflammation and cell survival 10-12. Deciphering these relationships is essential for uncovering the molecular basis of hematological malignancies and developing targeted therapeutic strategies. However, traditional statistical methods often fail to capture subtle associations, particularly in studies constrained by small sample sizes.

Clinical specimens, such as bone marrow (BM) aspirates, are typically obtained through invasive procedures. The necessity for patient consent and the challenging nature of sample collection often results in limited sample availability. To address these challenges and investigate the associations between *NF-κB1* and other cytokine genes in BM samples, machine learning (ML) and explainable artificial intelligence (XAI) techniques offer promising solutions 13-16. ML models, including k-Nearest Neighbors (kNN), Naïve Bayes Classifier (NBC), Random Forest (RF), and XGBoost, combined with XAI techniques such as SHapley Additive exPlanations (SHAP), can reveal significant patterns not evident through traditional statistical analyses. XAI provides critical insights into feature importance, enhancing interpretability and enabling researchers to identify influential genes and interactions.

Another challenge in rare disease research is identifying the minimum sample size required to achieve reliable classification accuracy. This can be addressed by employing adaptive ML techniques and systematically evaluating model performance across varying dataset sizes. Such an approach not only mitigates the limitations posed by data scarcity in rare diseases but also provides deep insights into NF-κB interactions.

By integrating ML and XAI, researchers can uncover complex biological relationships, offering new directions for precision medicine in hematological malignancies.

This study aimed to analyze *NF-κB1* and cytokine genes in bone marrow mononuclear cells in patients with hematological malignancies and to explore their interactions. The methodology integrates statistical analyses, dimensionality reduction techniques, and ML models to uncover complex gene interactions and validate findings, particularly in the context of small datasets.

## Materials and Methods

### Study population and data collection

Aliquots of leftover BM aspirate samples were collected from patients undergoing BM examinations for the diagnosis of hematological malignancies. Based on the world health organization diagnostic criteria, patients were classified into following groups after the BM smear and pathological review: myeloproliferative neoplasm (MPN), acute myeloid leukemia (AML), myelodysplastic syndrome (MDS), and plasma cell neoplasm (PCN). The control group (n = 17) comprised patients with lymphoma without BM involvement (n = 16) or normocellular marrow without hematological malignancy confirmed by BM smear and pathological review. BM aspirates were collected in BD Vacutainer tubes (Becton Dickinson, Franklin, NJ, USA) containing ethylene-diamine-tetra-acetic acid and centrifuged (2399 × *g* for 10 min). Following centrifugation, BM infranatants (including hematopoietic BM cells) were used for mononuclear cell (MNC) isolation. Baseline demographic information and hematological parameters, including hemoglobin levels, white blood cell (WBC) counts, neutrophil and platelet counts, C-reactive protein (CRP) levels, and estimated glomerular filtration rate (eGFR) were collected from medical records.

Lymphoprep (density gradient medium; density: 1.077 g/mL; STEMCELL Technologies, Vancouver, Canada) and SepMate tubes (STEMCELL Technologies, Vancouver, Canada) were used to isolate MNCs from BM aspirates. MNC isolation was performed as previously described (14). Isolated BM MNCs were stored at -80 °C until mRNA extraction. mRNA was extracted from BM MNCs as previously described (14) and RNA quality was determined using a 2100 Bioanalyzer capillary electrophoresis system (Agilent Technologies, Santa Clara, CA). Extracted RNA samples were run on an nCounter Analysis System (NanoString Technologies Inc., Seattle, WA, USA) following the manufacturer's instructions (14). A cytokine panel was used, which included the following genes (National Center for Biotechnology Information [NCBI] reference sequence): *BAX* (NM_138761.3)*, BCL2L1* (NM_138578.1)*, caspase-1* (NM_001223.3)*, INFB* (NM_002176.2)*, MyD88* (NM_002468.3)*, NF-κB1* (NM_003998.2)*, NFKB1A* (NM_020529.1)*, NGAL* (NM_005564.3)*, NLRP3* (NM_001079821.2)*, RAGE* (NM_014226.2), and* TNF* (NM_000594.2). *NF-κB1* (NM_003998.2) 17 encodes a 105 kD protein which can undergo cotranslational processing by the 26S proteasome to produce a 50 kD protein. The 105 kD protein is a Rel protein-specific transcription inhibitor and the 50 kD protein is a DNA binding subunit of the NF-κB protein complex.

To ensure the quality of the raw data, the following criteria were confirmed: (i) Imaging quality control (QC): Field of View >75%; (ii) Binding Density QC: 0.1 <Binding Density <2.25; (iii) Positive Control Linearity QC: R2 >0.95; and (iv) Limit of Detection QC: 0.5 fM positive control probe exceeded more than two standard deviation plus mean of the negative controls. The data were normalized using the control probes from Codeset. Normalized expression values of *NF-κB1* and ten other cytokine genes were used for the statistical analysis.

### Ethical considerations

This study was approved by the Institutional Review Board of Korea University Ansan Hospital (Ansan-si, Republic of Korea; Approval No. 2018AS0256), and was conducted in accordance with the principles outlined in the Declaration of Helsinki. Patients were recruited between May 2018 and July 2019, with all 114 participants provided informed consent.

### Study design

**Figure 2:**
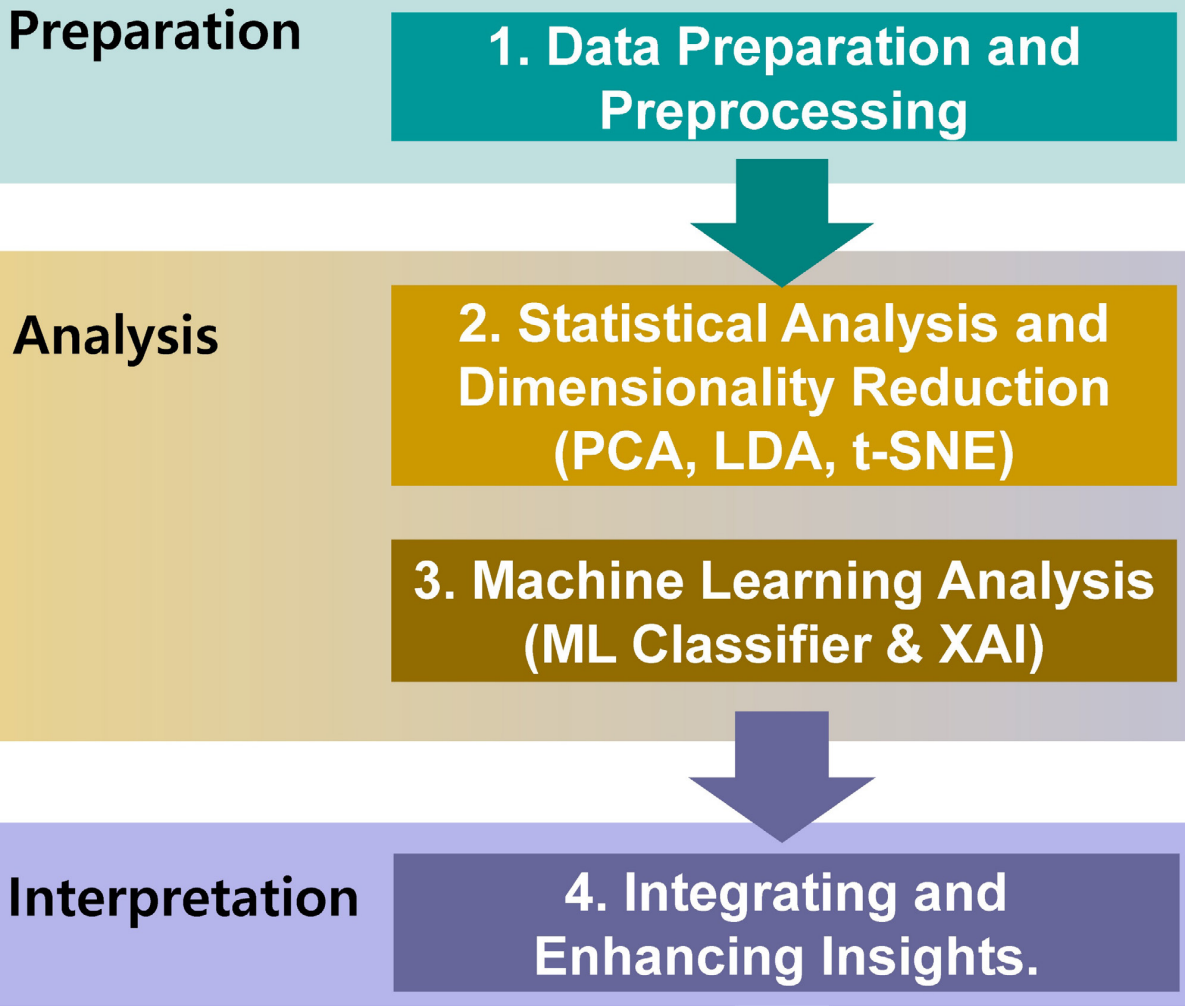
Flowchart of the study highlighting the three main phases: data preparation, analysis, and interpretation.

The flowchart (Figure 2) outlines a systematic framework for data analysis and interpretation, divided into three interconnected phases: data preparation, analysis, and interpretation.

In the data preparation phase, the dataset underwent preprocessing and normalization to ensure quality and compatibility for subsequent analyses. BM gene expression data were evaluated to preprocess key cytokine features, and scatter plots were generated to visualize initial feature distributions and relationships, such as *NF-κB1* gene expression vs. *caspase-1* gene expression, highlighting significant patterns. The analysis phase integrated statistical methods and ML techniques to extract deeper insights. Correlation analyses (Spearman, Kendall, and Pearson) identified relationships among cytokine features, such as caspase-1 and NGAL, while dimensionality reduction techniques—including principal component analysis (PCA), linear discriminant analysis (LDA), and t-distributed stochastic neighbor embedding (t-SNE)—were employed to simplify the dataset. PCA highlighted features contributing most to variance, LDA improved class separability, and t-SNE visualized non-linear clustering, providing complementary perspectives on data structure. Supervised ML models, including kNN, NBC, RF, and XGBoost, were applied to classify patient groups and assess feature importance. Explainable AI (XAI) methods, such as permutation feature importances and SHAP, were utilized to interpret model results. These methods revealed critical patterns, such as the strong association of *caspase-1* and *NF-κB1* gene expression in each hematological malignancy group—patterns that might have been overlooked by traditional statistical approaches.

In the interpretation phase, findings from statistical and AI-driven analyses were synthesized to enhance robustness and reliability. To address the challenges posed by small sample sizes in rare disease research, k-fold cross-validation was employed alongside systematic evaluations of dataset sizes to determine the minimum sample size needed for reliable classification. This approach optimized the utility of limited data and ensured robust model performance. Overall, this framework provided a deeper understanding of *NF-κB1* interactions, emphasizing their diagnostic and targeted therapeutic potential in hematological malignancies.

### Correlation analysis

Spearman correlation was selected as the primary method to evaluate monotonic relationships without assuming linearity or normal distribution 18, 19. To provide additional perspectives, complementary analyses using Kendall and Pearson correlations were also performed 20, 21. Spearman analysis revealed significant positive correlation between *NF-κB1* gene expression and expressions of *caspase-1*, *BAX*, and *NGAL* genes. In MDS and PCN groups, a notable positive correlation between *NF-κB1* and *caspase-1* gene expression suggested potential interactions relevant to disease progression. Consistent negative correlations between *NF-κB1* and *NGAL* gene expression across all patient groups indicated a potential inverse regulatory relationship.

### Dimensionality reduction

PCA was employed to reduce dataset dimensionality while preserving significant variance 22, 23. The first two principal components highlighted key variance-driven features. These components provided insights into patient clustering patterns and molecular-level differences between the groups. LDA further optimized class separability by identifying feature combinations that enhanced group discrimination 24, 25. Non-linear relationships were explored using t-SNE, which effectively visualized clustering and sub-group patterns in two dimensions 26.

### ML models for classification of disease groups

Beyond conventional statistical analyses and dimensionality reduction methods, ML techniques were employed to analyze *NF-κB1* gene expression-related profiles and classify disease groups with high accuracy. By leveraging ML classifiers, we aimed not only to achieve robust predictive performance but also to gain insight into the key features that distinguish each disease subgroup, even when working with relatively small datasets—a common challenge in biomedical research 27, 28. Demonstrating reliable classification under these constraints lends credibility to our findings and supports the feasibility of similar studies with restricted sample availability.

Specifically, gene expression data were normalized using z-score transformation to standardize the dataset, and features were selected based on variance. Four classifiers—k-Nearest Neighbors (kNN) 29, Naïve Bayes Classifier (NBC) 30, Random Forest (RF) 31, and XGBoost 13— were implemented to classify disease groups. The dataset was divided into training and testing sets using an 80-20 split, and model evaluation was conducted using k-fold cross-validation (k = 3) to ensure robust and unbiased estimation of performance metrics. Key metrics assessed for each model included classification accuracy, precision, recall, and F1-score, thereby enabling a comprehensive assessment of predictive performance.

To identify features pivotal for group discrimination, permutation feature importances were analyzed. Notably, the classifiers maintained high accuracy despite the limited sample size, underscoring the potential of ML-based methodologies in rare disease research. This observation motivated further analyses on minimal sample requirements, confirming that the models' robustness and predictive power could be retained even under more stringent data constraints. Therefore, the use of ML classification frameworks complements traditional analytical methods, providing both strong predictive accuracy and biologically meaningful feature selection.

### XAI regression analysis of NF-κB relationships

To explore relationships between *NF-κB1* gene expression and other key features, we employed XGBoost regression and used SHAP to enhance interpretability by quantifying the contribution of each feature to model predictions 32. SHAP summary plots identified key features, including *caspase-1*, *RAGE*, *NGAL*, *MYD88*, and *BCL2L1* normalized counts, underscoring their roles in inflammation and apoptosis pathways. k-fold cross-validation (k = 3) with a 10% test size ensured robust performance, balancing effective training and validation for small datasets. This approach offered a transparent exploration of *NF-κB1* interactions, advancing the understanding of its regulatory pathways in hematological malignancies.

### Small sample size adaptation

To evaluate the robustness of classification performance across varying sample sizes, we tested models on combined sample sizes that included each disease group (MPN, AML, MDS, PCN) and the control group. The total sample size was systematically increased, ranging from five to the maximum available samples for each disease and its corresponding control group. An exception was made for kNN, which required a minimum of eight samples due to its reliance on nearest neighbor calculations. The goal was to determine the minimum sample size required to achieve reliable and meaningful classification results, defined as accuracy ≥ 80-90%. k-fold cross-validation (k = 3) was employed to ensure consistent and robust performance across all sample sizes. This approach demonstrated that for AML and MDS, reliable classification accuracy was achieved with as few as 15 and 14 samples, respectively. In contrast, MPN and PCN exhibited greater variability in performance, requiring larger sample sizes yet exhibiting instability, even at higher thresholds. These findings provide valuable insights into the minimum dataset requirements for effective classification, addressing a critical challenge in small data scenarios characteristic of rare disease research. By incrementally increasing the sample size, this analysis revealed the thresholds at which the ML models—kNN, NBC, RF, and XGBoost—achieved stable and reliable accuracy when distinguishing between disease groups and the control group.

This approach provides insight into the sample size requirements for efficient machine learning-based categorization, which may help guide future investigations with comparable restricted data. It illustrates that, while smaller datasets can still produce reliable results for certain disease categories (AML and MDS), bigger datasets may be required to get consistent performance in others (MPN and PCN), especially where biological complexity or heterogeneity are high.

## Results

### Patient characteristics

BM aspirates were collected from 114 patients with a median age of 63 (range 22-90) years. All 114 patients were at the initial diagnosis stage. Patient demographics, hematological parameters, and *NF-κB1* gene normalized counts are shown in Table 1.

As for the MPN, AML, MDS, PCN, and control groups, patient demographics and laboratory parameters are presented in Table 2. Ten continuous variables exhibited significant differences among the five groups (Table 2).

Among them, six laboratory parameters (except for *NF-κB1*, *caspase-1*, *BAX*, and *NGAL* normalized counts) exhibited the following intergroup differences (Table 3): Hb and neutrophil counts were significantly lower in the AML and MDS groups than those in the MPN and control groups. Furthermore, the Hb count was significantly lower in the PCN group than that in the MPN group. WBC counts were significantly lower in the AML and MDS groups than those in the MPN group, being significantly lower in the MDS group than those in the control group. Platelet counts were significantly lower in the AML, MDS, PCN, and control groups than those in the MPN group. The CRP was significantly higher in the AML group than that in the MPN group. eGFR was significantly lower in the PCN group than that in the MPN and control groups.

**Figure 3:**
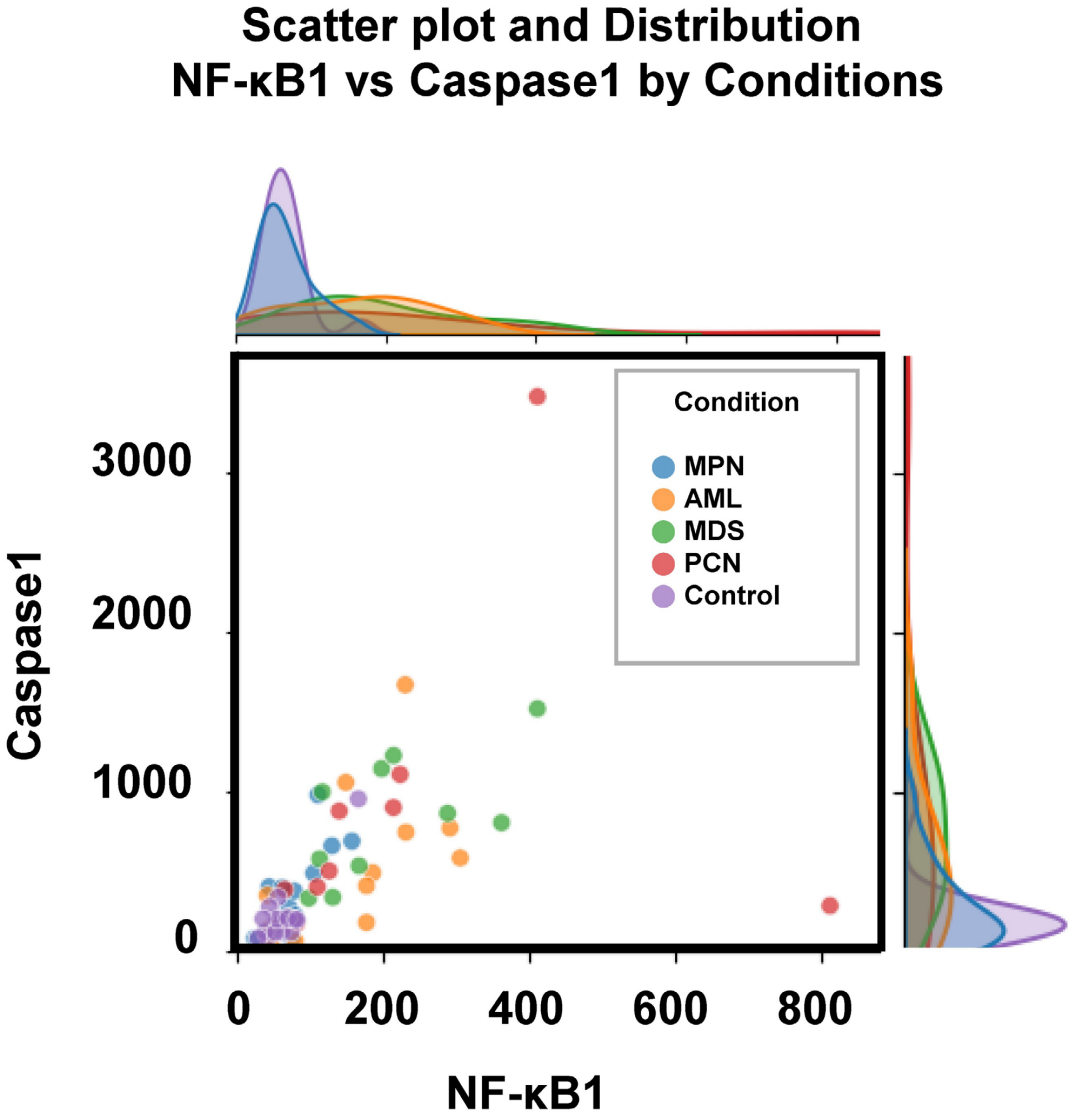
Scatter plot and marginal density distributions of *caspase-1* and *NF-κB1* normalized counts across the five patient groups (MPN, AML, MDS, PCN, and Control). The scatter plot highlights intergroup differences, while the marginal density plots provide an overview of the parameter distributions within each group, emphasizing the overlapping trends and variability among the groups. **Abbreviations**: AML, acute myeloid leukemia; MDS, myelodysplastic syndrome; MPN, myeloproliferative neoplasm; PCN, plasma cell neoplasm

Figure 3 depicts the distribution of two key parameters, *caspase-1* and *NF-κB1* normalized counts, across the five groups, highlighting the observed differences outlined in Table 3. These parameters were selected for scatter plot analysis due to their pivotal roles in regulating inflammation and apoptosis—processes critically involved in the pathogenesis of hematological malignancies 12, 33. Caspase-1 is a key mediator of the inflammatory response, activating pro-inflammatory cytokines such as IL-1β and IL-18, and playing a pivotal role in cell death pathways, which are often dysregulated in cancer 9, 34. Also, the *NFKB1* gene displays pivotal roles in the regulation of inflammatory responses and genetic variations in this gene have been documented in the several pathologies 9, 35. Scatter plot analysis of these parameters provided an initial exploration of their group-wise distributions, offering insights into their potential interactions. Significant group-specific differences were observed in *caspase-1* and *NF-κB1* normalized counts; however, overlaps among groups such as AML, MDS, and PCN highlighted the complexity of these interactions. This observed overlap highlights the inherent limitations of conventional statistical analyses and standard visualization techniques in distinguishing subtle group differences within high-dimensional datasets.

### Correlation analysis

**Figure 4:**
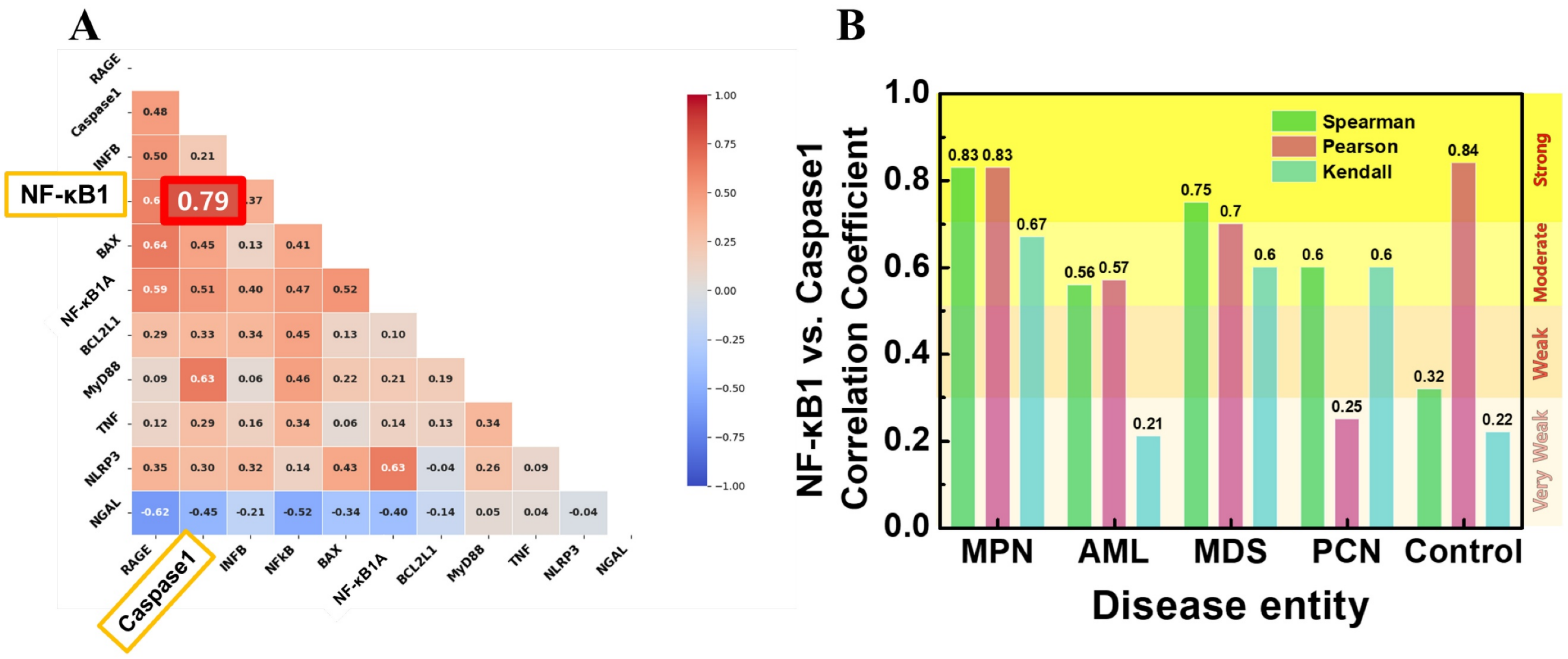
(A) Pearson correlation heatmap illustrating the relationships between *NF-κB1* and other cytokine genes. *Caspase-1* normalized counts demonstrates the strongest correlation with *NF-κB1* normalized counts (r = 0.79). (B) Correlation coefficients (Spearman, Pearson, and Kendall) between *NF-κB1* and *caspase-1* normalized counts across the five patient groups. The MPN group shows consistently strong correlations across all methods, highlighting the robust interaction between these two features.

To investigate the relationships between expression of *NF-κB1* and other key cytokine genes, correlation analyses were conducted. Figure 4A presents a Pearson correlation heatmap, illustrating the strength and direction of relationships across selected features. Among the analyzed variables, *caspase-1* normalized counts exhibited the strongest positive correlation with *NF-κB* normalized counts (r = 0.79), indicating a strong association between these two genes. Figure 4B shows the correlation coefficients between *NF-κB1* and *caspase-1* normalized counts using three different methods—Spearman, Pearson, and Kendall—across each patient group. In the MPN group, *caspase-1* and *NF-κB1* gene normalized counts demonstrated consistently high correlation coefficients, with Pearson's r = 0.84, Spearman's r = 0.83, and Kendall's τ = 0.83. These strong correlations suggest a central role for caspase-1 in regulating NF-κB activity within the MPN group. By contrast, the control group exhibited moderate correlations, with Pearson's r = 0.56, Spearman's r = 0.57, and Kendall's τ = 0.32, indicating weaker co-regulation between *caspase-1* and *NF-κB1* normalized counts in non-malignant conditions. In the AML and MDS groups, correlations were moderate, with Pearson's r values ranging between 0.67 and 0.70, reflecting the inflammatory activity commonly associated with these malignancies. The PCN group showed weak correlations across all methods, with the highest Pearson value at r = 0.60, suggesting a less direct relationship in this group.

### Statistical analyses (Simple, Multiple regression and PCA, LDA, t-SNE)

*NF-κB1* normalized counts (median [Q1, Q3]) in BM MNCs (n = 114) were 87.78 (57.06, 172.50) (Table 1). Among ten cytokine genes, a simple regression analysis identified six significant predictors of *NF-κB1* normalized counts (Table 4). Table 4 describes the normalized counts (median [Q1, Q3]) of each of the six cytokine genes.

Using normalized counts of* NF-κB1* as the dependent variable and six significant predictors (normalized counts of six cytokine genes) as independent variables, a multiple regression analysis was performed. The following multiple regression models were developed (Table 5).

(Model) *NF-κB1* normalized counts = 75.034 + 0.157 × *caspase-1* normalized counts + 0.03 × *BAX* normalized counts - 0.001 × *NGAL* normalized counts (Adjusted R^2^ = 0.434, *P* <0.0001)

**Figure 5:**
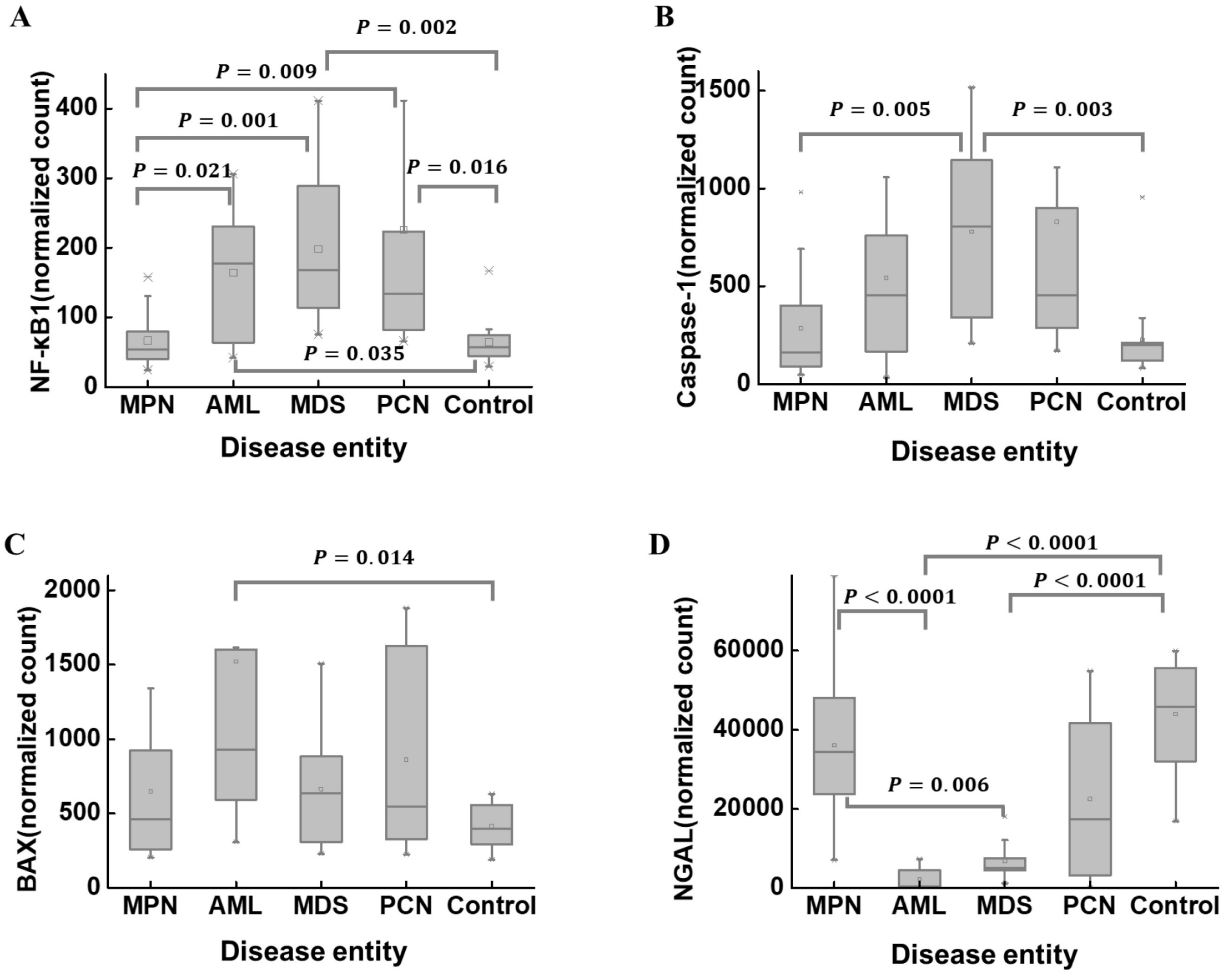
A comparison of the normalized counts of *NF-κB1* (A), *caspase-1* (B), *BAX* (C), and *NGAL* (D) in BM mononuclear cells of the hematological malignancy and control groups. The control group comprises patients with normal BM. (A) NF-κB1 normalized counts in the MPN and control groups are statistically lower than those in the AML, MDS, and PCN groups. (B) *Caspase-1* normalized counts in the MPN and control groups are statistically lower than those in the MDS group. (C) BAX normalized counts in the control group are statistically lower than those in the AML group. (D) NGAL normalized counts in the AML and MDS groups are statistically lower than those in the MPN and control groups. **Abbreviations**: AML, acute myeloid leukemia; BAX, BCL2-associated X; BM, bone marrow; MDS, myelodysplastic syndrome; MPN, myeloproliferative neoplasm; NF-κB1, nuclear factor kappa light chain enhancer of activated B cells 1; NGAL, neutrophil gelatinase-associated lipocalin; PCN, plasma cell neoplasm.

The median (Q1, Q3) of *NF-κB1* normalized counts according to disease entities are summarized in Table 2. *NF-κB1* normalized count in the control group was 56.75 (43.55, 76.98). The MPN group (n = 20) exhibited the lowest *NF-κB1* normalized count (54.25 [39.21, 79.56]). The MPN and control groups exhibited statistically lower *NF-κB1* normalized counts than those in the AML, MDS, and PCN groups (Figure 5A). The median (Q1, Q3) of *caspase-1* normalized counts in the control group was 199.82 (120.76, 213.35) (Table 2). The MDS group (n = 11) exhibited the highest *caspase-1* normalized counts (805.92 [339.01, 1144.15]). The MDS group exhibited statistically higher *caspase-1* normalized counts those that in the MPN and control groups (Figure 5B). The median (Q1, Q3) of *BAX* normalized counts in the control group was 396.47 (292.88, 559.31) (Table 2). The AML group exhibited statistically higher *BAX* normalized counts than those in the control group (Figure 5C). The median (Q1, Q3) of *NGAL* normalized counts in the control group was 45859.73 (31338.67, 55617.08) (Table 2). The AML group exhibited statistically lower *NGAL* normalized counts than those in the MPN and control groups (Figure 5D). The MDS group exhibited statistically lower *NGAL* normalized counts than those in the MPN and control groups (Figure 5D). These results further support the previously observed correlation between *NF-κB1* and *caspase-1* normalized counts (Figure 4), as their group-wise distribution trends align closely (Figure 5A, B), suggesting a potential interdependence between these features.

**Figure 6:**
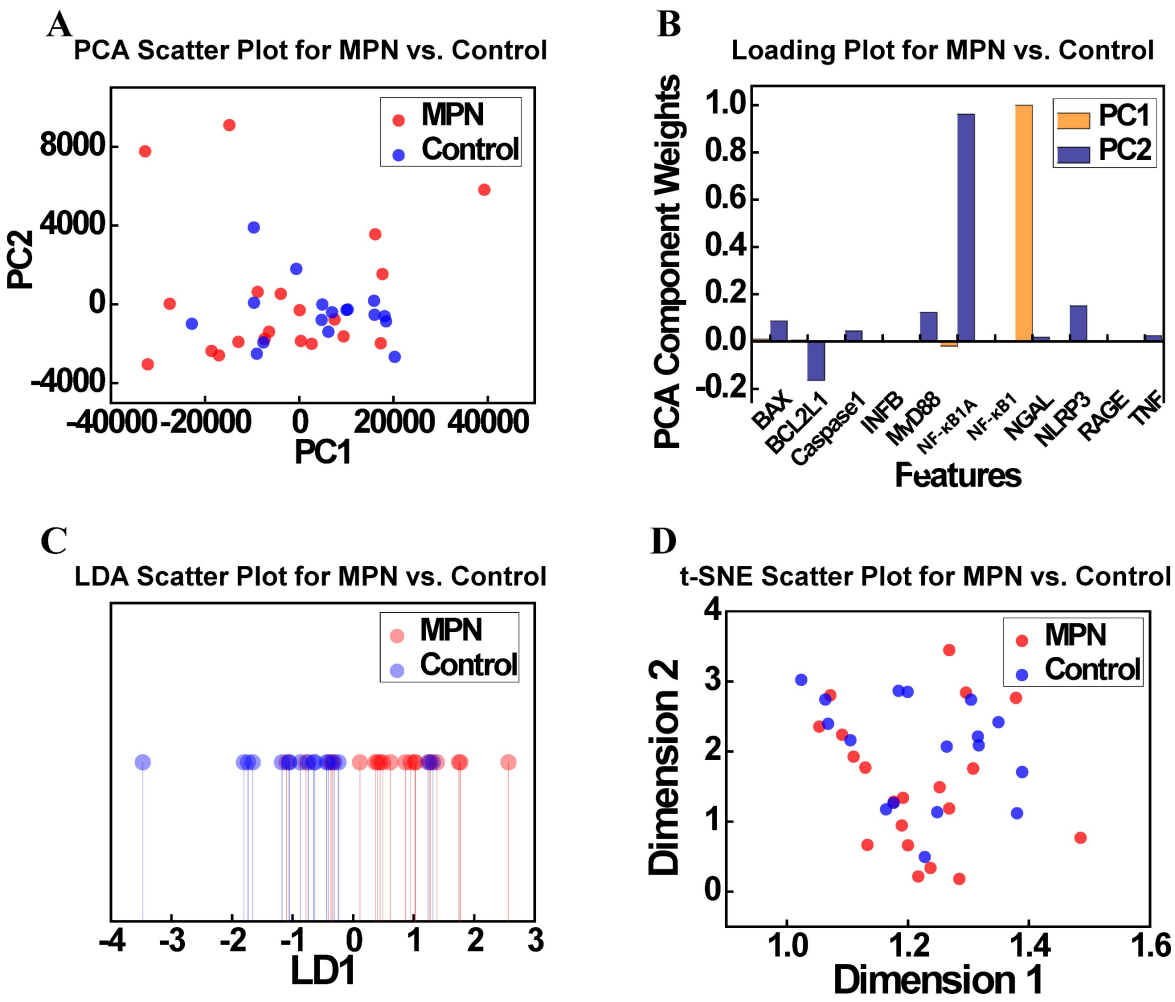
(A) PCA scatter plot showing the distribution of the MPN and Control groups along the first two principal components (PC1 and PC2). (B) PCA loadings indicating the contributions of key features (e.g., *NF-κB1* normalized counts, *caspase-1* normalized counts) to PC1 and PC2. (C) t-SNE scatter plot depicting the clustering of MPN and Control groups in a non-linear feature space. (D) Loading projections highlighting the most influential features in the t-SNE analysis. **Abbreviations:** MPN, myeloproliferative neoplasm; PCA, principal component analysis; PC, principal component; LDA, linear discriminant analysis; t-SNE, t-distributed stochastic neighbor embedding; LD, linear discriminant.

To further explore the relationships identified in the correlation analysis (Figure 4), dimensionality reduction techniques were applied to the MPN and Control groups. Figure 6 presents the results of PCA and t-SNE, offering insights into the separability of these groups based on gene expression profiles. Figure 6A illustrates the PCA scatter plot showing the distribution of the MPN and Control groups along the first two principal components (PC1 and PC2). Although some degree of separation is observed, significant overlap persists, suggesting that PCA alone cannot fully resolve the complex relationships between these groups. Figure 6B displays the PCA loadings plot, identifying the contributions of features to the principal components. Contrary to expectations, *NF-κB1* and *caspase-1* normalized counts were not dominant contributors to PC1 or PC2, with other features emerging as more influential. This highlights the limitations of PCA in isolating key biological drivers within complex and high-dimensional datasets. Figure 6C presents the t-SNE scatter plot, a non-linear dimensionality reduction method, which provides an alternative view of local clustering patterns. Although t-SNE reveals a slight separation between MPN and Control groups, substantial overlap remains, mirroring the findings from PCA. Figure 6D, which visualizes the t-SNE feature loadings and similarly does not identify *NF-κB1* and* caspase-1* normalized counts as major contributors to clustering patterns, further highlighting the challenges of distinguishing these groups using these methods. Taken together, these findings demonstrate the limitations of dimensionality reduction techniques—both linear (PCA) and non-linear (t-SNE)—in fully resolving the subtle molecular distinctions between MPN and Control groups.

To further support these observations, additional analyses were performed for the AML, MDS, and PCN groups as presented in Supplementary Figures S1-S3, providing a broader perspective on group separability. For AML and MDS, clearer clustering patterns emerged, particularly in LDA plots, highlighting stronger differentiation relative to Control groups. Conversely, the PCN group exhibited substantial overlap with the Control group across all methods, reflecting the molecular complexity and weaker discriminatory signals within this group. These supplementary results provide additional evidence of disease-specific molecular patterns and emphasize the importance of multi-method approaches in identifying subtle gene expression differences.

### ML classification

**Figure 7:**
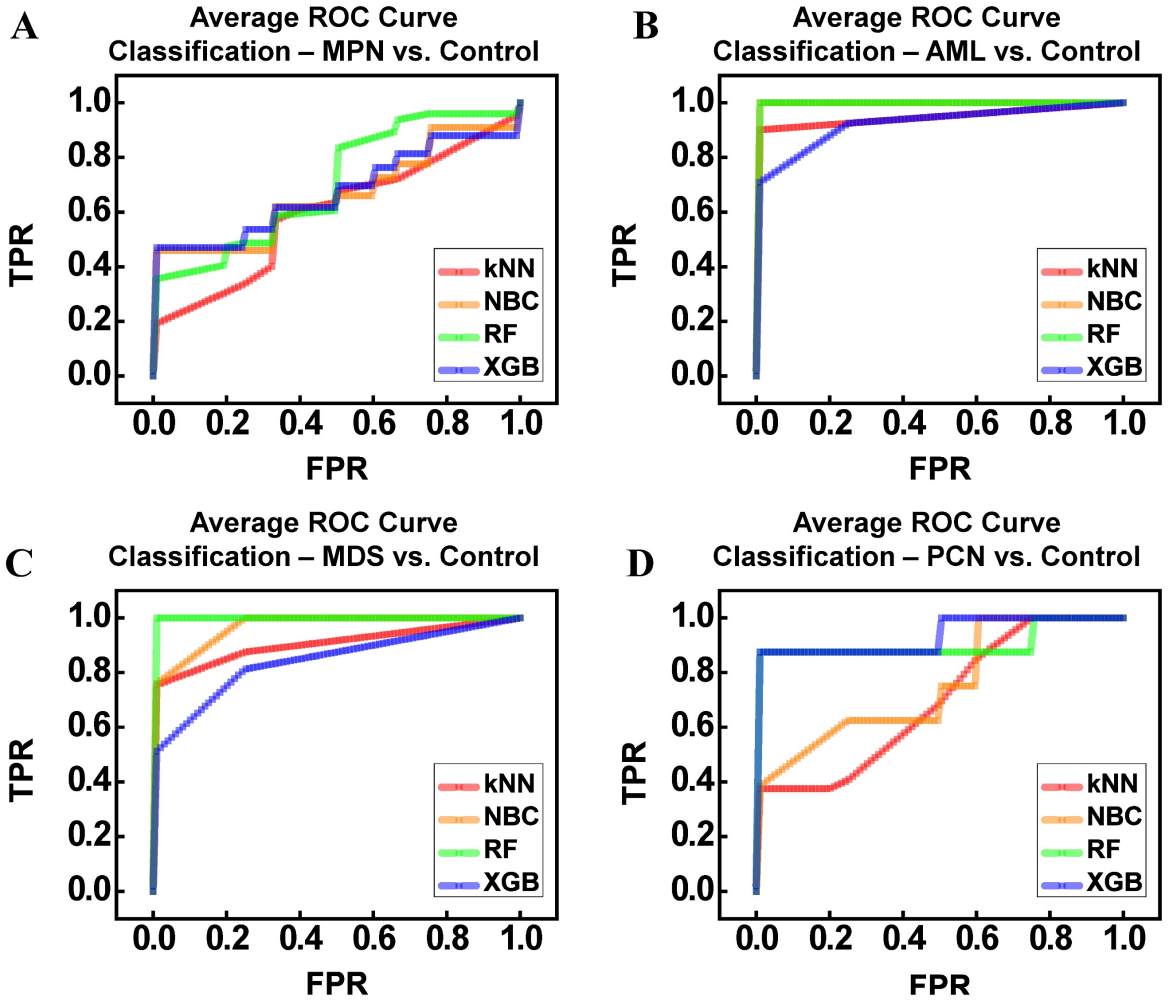
Average ROC Curves for Machine Learning Classifiers (kNN, NBC, RF, XGB) Applied to Distinguish Between Control and Hematological Malignancy Groups. (A) MPN vs Control. (B) AML vs Control. (C) MDS vs Control. (D) PCN vs Control. **Abbreviations:** MPN, myeloproliferative neoplasm; AML, acute myeloid leukemia; MDS, myelodysplastic syndrome; PCN, plasma cell neoplasm; ROC, receiver operating characteristic; TPR, true positive rate; FPR, false positive rate.

The average receiver operating characteristic (ROC) curves in Figure 7 illustrate the classification performance of ML models—kNN, NBC, RF, and XGBoost (XGB)—across comparisons between disease groups (MPN, AML, MDS, PCN) and the control group. To ensure robust and unbiased evaluation, we employed k-fold cross-validation (k = 3), systematically training and testing the models across different subsets of the data. The reported performance metrics, including accuracy, precision, recall, and area under the ROC curve (AUC), reflect the average values obtained across all folds. This approach minimizes bias and variance, which are common challenges in small datasets, enhancing confidence in the results 36-38. Figure 7A illustrates the ROC curves for distinguishing between the MPN and control groups. Among the classifiers, RF (AUC = 0.81) demonstrated the highest performance, followed by NBC (AUC = 0.66) and XGB (AUC = 0.67). In contrast, kNN demonstrated the lowest performance (AUC = 0.60), likely due to its sensitivity to local data structures and inability to capture complex patterns in high-dimensional, small datasets. Despite RF's relatively superior results, the moderate accuracy (62%) and F1-score (67%) indicate substantial molecular overlap between MPN and control groups, a finding consistent with earlier dimensionality reduction analyses (Figure 6). Figure 7B shows the ROC curves for AML vs control classification. All models performed strongly, with NBC and RF achieving perfect classification (AUC = 1.00), while XGB achieved comparably high performance (AUC = 0.93). The kNN model also performed well (AUC = 0.95). Precision, recall, and F1-scores further confirmed these results, reflecting the distinct gene expression patterns observed in AML, which facilitated clear separation from the control group. Figure 7C focuses on the ROC curves for distinguishing between MDS and control groups. Both NBC (AUC = 0.97) and RF (AUC = 1.00) demonstrated excellent performance, while XGB achieved a slightly lower AUC (0.84). The kNN model performed reasonably well (AUC = 0.91). However, variability in precision and F1-scores (ranging 61-90%) highlights the subtle molecular overlaps between MDS and control samples, posing challenges for consistent classification despite the overall high AUC values. Figure 7D presents the ROC curves for PCN vs control classification. RF (AUC = 0.91) and XGB (AUC = 0.94) outperformed simpler models like NBC (AUC = 0.76) and kNN (AUC = 0.70). While ensemble methods performed well, the relatively lower F1-scores (RF = 65%, XGB = 63%) suggest significant gene expression overlap between PCN and control groups, reflecting the molecular complexity of PCN. Overall, Table 6 summarizes the model performance metrics—accuracy, precision, recall, F1-score, and AUC—across all disease group comparisons. RF consistently demonstrated the highest performance, achieving the best AUC values for MPN (0.81), AML (1.00), MDS (1.00), and PCN (0.91). XGB also performed strongly, particularly in PCN (AUC = 0.94). NBC excelled in AML (AUC = 1.00) and MDS (AUC = 0.97), underscoring its suitability for small, high-dimensional datasets. In contrast, kNN exhibited variable performance, achieving strong results in AML but performing poorly in MPN (AUC = 0.60) and PCN (AUC = 0.70). These findings highlight the strengths of ensemble models (RF and XGB) in capturing non-linear patterns and relationships, while simpler models like kNN were limited by their reliance on local data distributions. Importantly, the use of k-fold cross-validation and averaging across all subsets ensured that these results were robust and reproducible. The consistent performance of ensemble methods across folds further emphasizes their reliability in addressing challenges posed by small sample sizes and overlapping gene expression patterns, making them particularly suitable for rare disease research.

### Permutation feature importance for classifying control and disease groups across ML models

**Figure 8:**
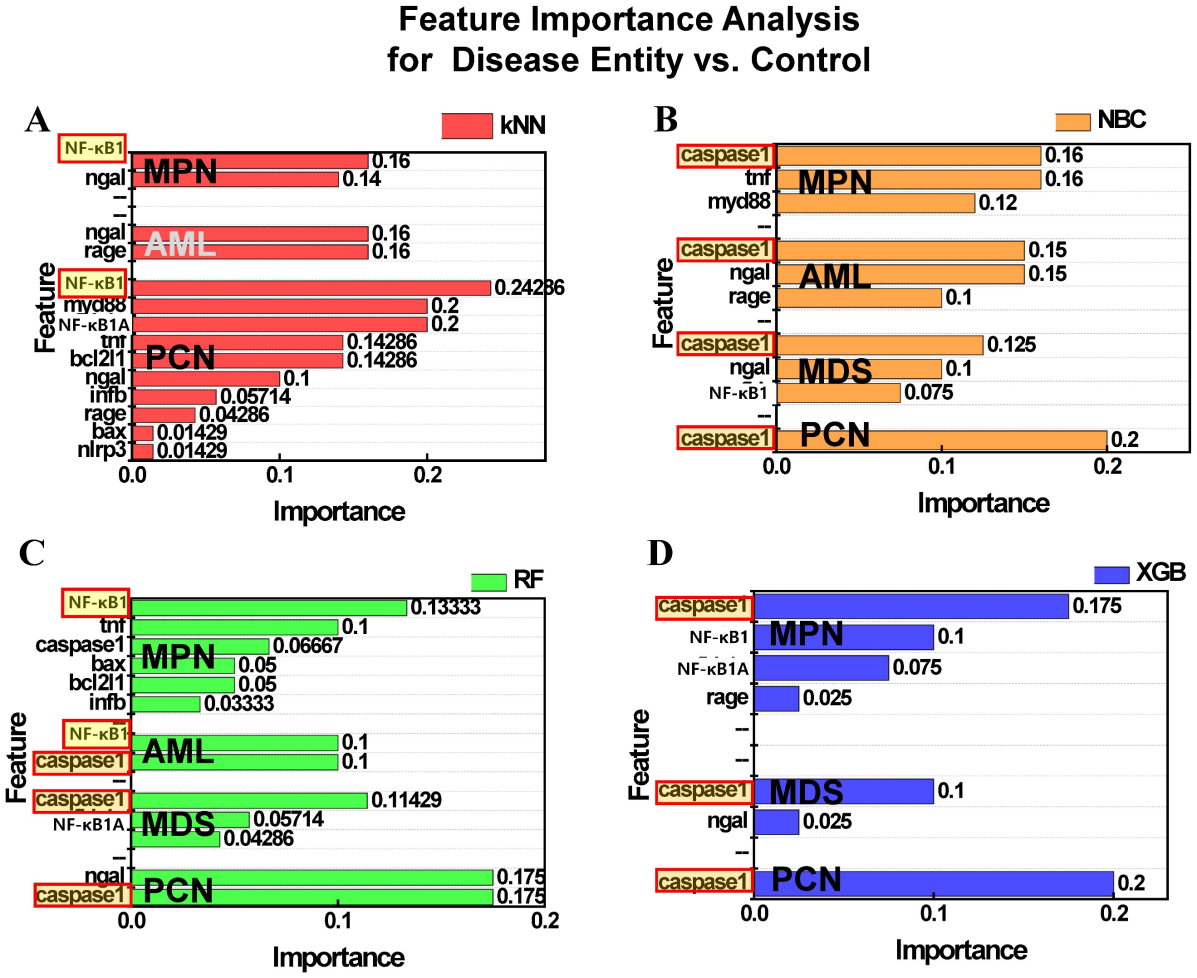
Permutation feature importance for distinguishing control and disease groups (MPN, AML, MDS, PCN) across four machine learning models: (A) kNN, (B) NBC, (C) RF, and (D) XGB. Bars represent the relative feature importance of *NF-κB1* and *caspase-1* normalized counts for classification tasks. Features highlighted with red-bordered yellow boxes indicate the top feature within each group that contributed the most to classification. Absence of bars (e.g., XGB for AML) reflects models where no features contributed relevantly to classification in the corresponding group. **Abbreviations:** MPN, myeloproliferative neoplasm; AML, acute myeloid leukemia; MDS, myelodysplastic syndrome; PCN, plasma cell neoplasm; kNN, k-nearest neighbors; NBC, naïve Bayes classifier; XGB, extreme gradient boosting; NF-κB1, nuclear factor kappa light chain enhancer of activated B cells 1; Caspase-1, cysteine-aspartic protease 1.

Figure 8 presents the results of permutation feature importance analysis across the four ML models. Figure 8A illustrates the feature importance as determined by the kNN model. For both MPN and PCN groups, *NF-κB1* normalized counts demonstrated the highest contribution to classification, emphasizing its significance in distinguishing these groups. However, for AML and MDS, the kNN model failed to identify significant features, reflecting the algorithm's inherent simplicity and limited ability to capture complex patterns within these datasets. Figure 8B displays the results from the NBC, where caspase-1 consistently emerged as the most influential feature across all groups—MPN, AML, MDS, and PCN. This highlights the robustness of NBC in identifying a universal feature with strong predictive relevance for group differentiation. Figure 8C highlights the feature importance derived from the RF model. For MPN, *NF-κB1* normalized counts were identified as the most critical feature, reflecting its dominant role in this group. In contrast, AML showed a co-dominance of *NF-κB1* and *caspase-1* normalized counts, suggesting a combined regulatory influence. For MDS and PCN, caspase-1 emerged as the most important feature, underscoring its pivotal role in these groups' inflammatory and apoptotic pathways 9, 34. Figure 8D presents the results of the XGB model, which provided relevant insights for MPN, MDS, and PCN groups. For all three groups, *caspase-1* normalized counts were identified as the dominant feature, further reinforcing its universal relevance across these malignancies. However, no significant feature importance was observed for AML, likely due to data imbalance or insufficient representation of key patterns within this group.

### Regression analysis and interpretability

**Figure 9:**
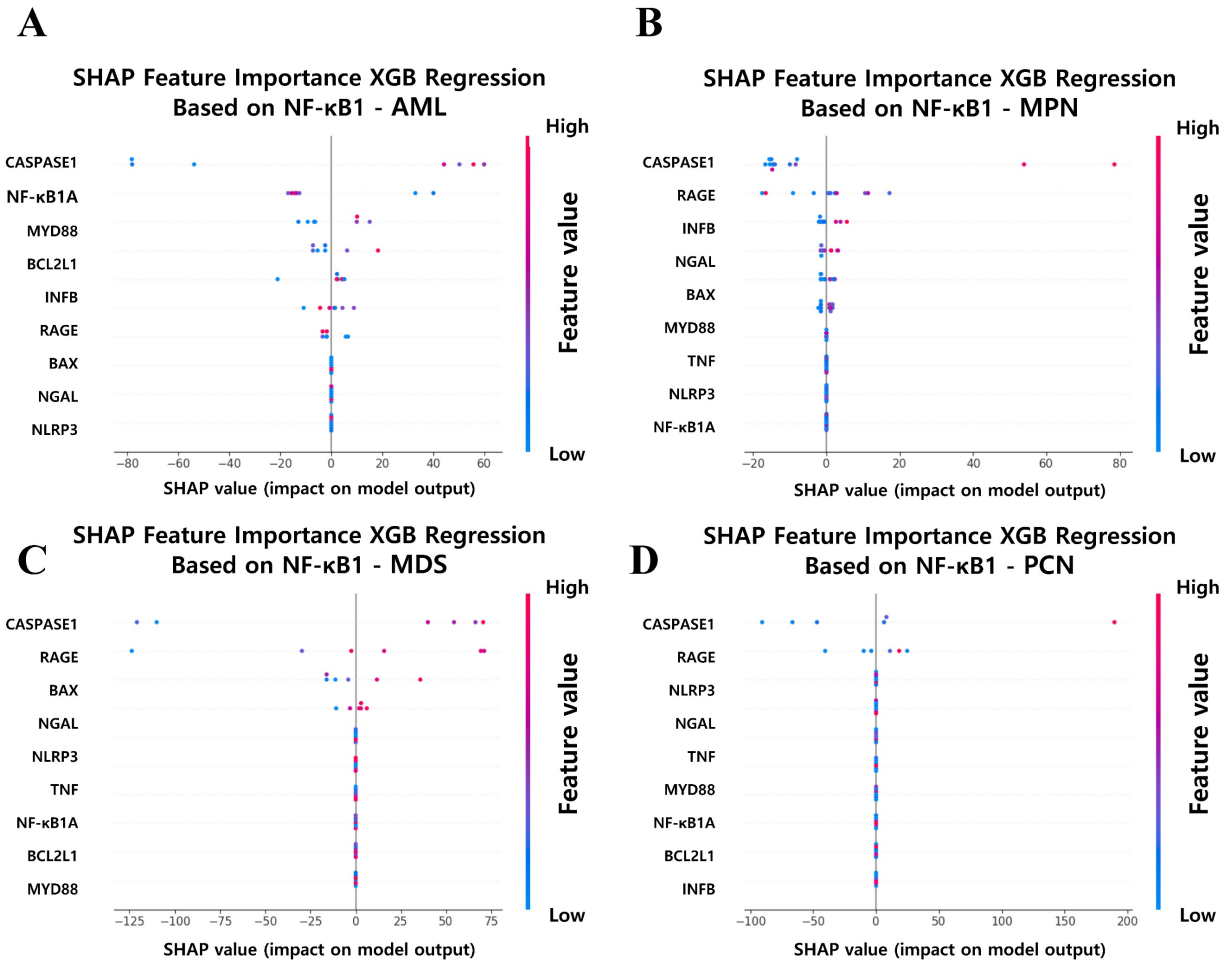
SHAP Feature Importance Analysis for Predicting NF-κB1 Expression Across Hematological Malignancy Groups. (A) AML, (B) MPN, (C) MDS, and (D) PCN groups. Caspase-1 consistently ranks as the top feature in all groups, underscoring its critical role in NF-κB1 regulation. Group-specific features, such as RAGE, NGAL, and MYD88, further highlight distinct regulatory mechanisms contributing to NF-κB1 activity across malignancy types. Abbreviations: MPN, myeloproliferative neoplasm; AML, acute myeloid leukemia; MDS, myelodysplastic syndrome; PCN, plasma cell neoplasm; XGB, extreme gradient boosting; Caspase-1, cysteine-aspartic protease 1; SHAP, SHapley Additive exPlanations.

To further elucidate the key features of driving *NF-κB1* normalized counts across hematological malignancy groups, SHAP analysis was applied to the XGB regression models. This approach enabled the identification and quantification of each feature's impact on *NF-κB1* normalized counts, providing a transparent and interpretable framework for understanding the molecular determinants of *NF-κB1* gene activity. Figure 9 illustrates the SHAP-based feature importance derived from XGB regression models for predicting *NF-κB1* normalized counts in the four hematological malignancy groups: AML, MPN, MDS, and PCN. Across all groups, *caspase-1* gene normalized counts consistently emerged as the most influential feature, reinforcing its central role in the regulation of NF-κB1-mediated inflammatory pathways. This finding suggests that *caspase-1* gene normalized counts represents a universal driver of *NF-κB1* gene activity and a potential therapeutic target for modulating inflammatory responses in various hematological malignancies. Beyond *caspase-1* gene normalized counts, the SHAP analysis highlights group-specific features, reflecting the unique regulatory mechanisms in each malignancy type. In the AML group (Figure 9A), identified were notable contributions from *NFKB1A*, *MYD88*, and *BCL2L1* normalized counts. The influence of *NFKB1A* gene normalized counts is consistent with the previous report that NF-κB activity would be influenced by NFKBIA, which​ ​encodes​ ​the​ ​main​ ​NFKB​ ​inhibitor,​ ​I​ ​kappa​ ​B​ ​alpha 39. *MYD88* gene highlights immune signaling pathways, and *BCL2L1* gene emphasizes the role of anti-apoptotic pathways in modulating the *NF-κB1* gene activity. These findings underscore the multifaceted nature of NF-κB regulation in AML pathophysiology, characterized by a complex interplay of immune and apoptotic factors. For the MPN group (Figure 9B), features such as *RAGE*, *INFβ*, and *NGAL* normalized counts played significant roles alongside caspase-1, indicating the involvement of receptor-mediated signaling and immune modulation in driving *NF-κB1* gene activity. In the MDS group (Figure 9C), *RAGE* and *BAX* normalized counts emerged as key features. The prominence of *RAGE* gene normalized counts suggests that inflammatory signaling is a primary driver of *NF-κB1* gene activity, while *BAX* gene indicates the simultaneous regulation of apoptosis in MDS. This dual influence highlights the delicate balance between inflammation and programmed cell death in the pathophysiology of MDS. Finally, in the PCN group (Figure 9D), *RAGE* normalized counts were identified as significant contributors, suggesting that inflammatory and immune responses play dominant roles in *NF-κB1* gene regulation in plasma cell neoplasms. The consistent importance of these features underscores the centrality of inflammation in PCN pathogenesis.

**Figure 10:**
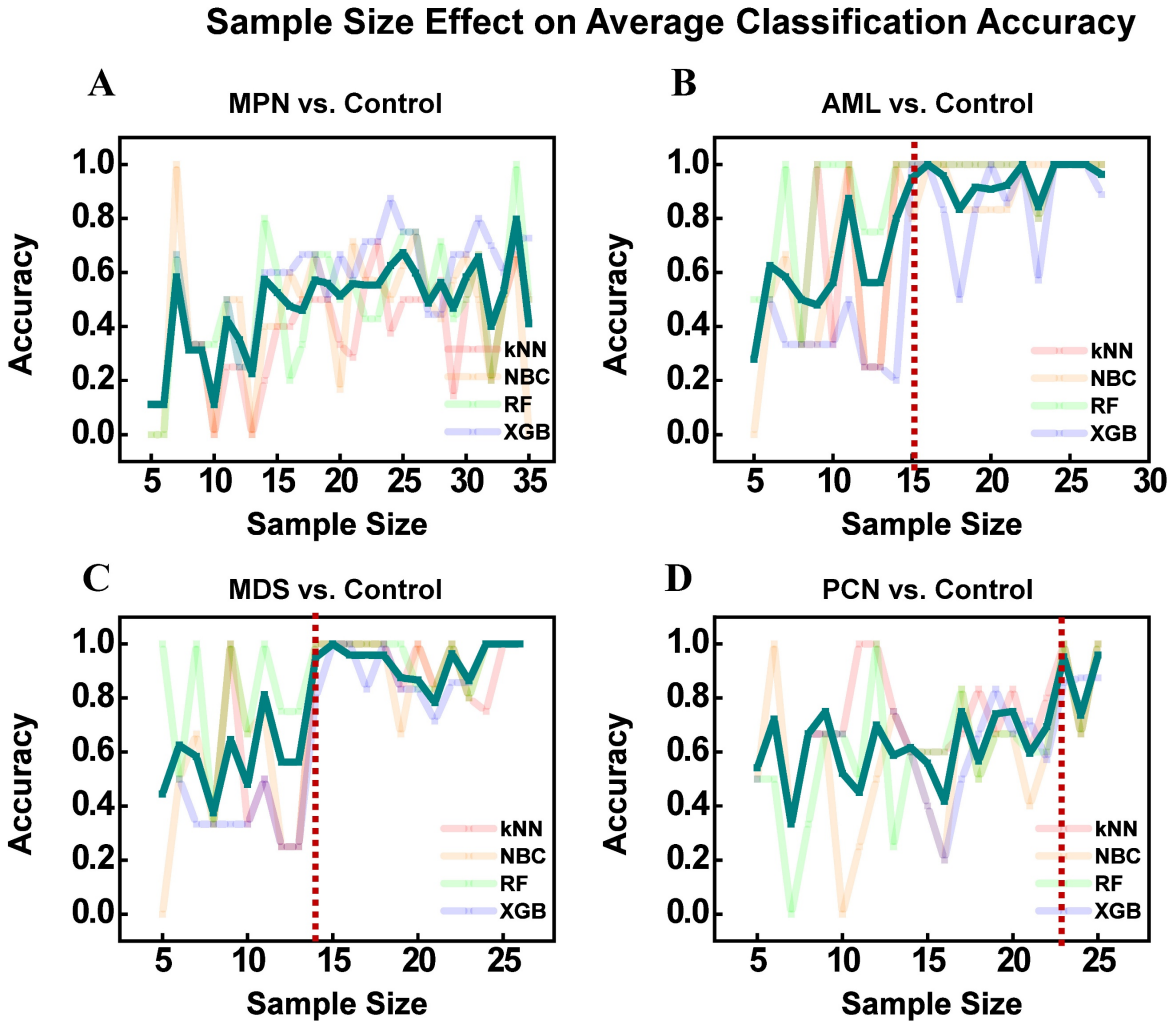
Average classification accuracy of four machine learning models (kNN, NBC, RF, and XGB) across varying sample sizes for distinguishing disease groups from the control group. (A) MPN, (B) AML, (C) MDS, and (D) PCN. The red dotted line indicates the sample size threshold (15, 14, and 23 samples) at which stable accuracy is achieved across most models for each disease group. Abbreviations: MPN, myeloproliferative neoplasm; AML, acute myeloid leukemia; MDS, myelodysplastic syndrome; PCN, plasma cell neoplasm; kNN, k-nearest neighbors; NBC, naïve Bayes classifier; XGB, extreme gradient boosting.

To evaluate the reliability of ML performance on small datasets and determine the minimum sample size required for stable classification accuracy, systematic analyses were conducted to measure the average classification performance of four ML models—kNN, NBC, RF, and XGB—across varying sample sizes for each disease group (AML, MPN, MDS, and PCN) against the control group. The results, illustrated in Figure 10, provide insights into the relationship between sample size and average classification accuracy, emphasizing the strengths and limitations of ML models when applied to small datasets in rare disease research. For AML vs. Control and MDS vs. Control, the average accuracy across the four ML models improved steadily with increasing sample sizes. Beyond thresholds of 15 and 14 samples for AML and MDS, respectively, the performance of RF and XGB stabilized consistently above 0.8, indicating strong and reliable classification accuracy. In contrast, simpler models like kNN and NBC exhibited greater sensitivity to smaller sample sizes, with accuracy fluctuating initially, before improving progressively. This pattern suggests that while kNN and NBC demonstrate rapid gains in performance as sample sizes increase, their variability highlights a reliance on larger datasets to achieve stable results compared to the more robust performance of ensemble models like RF and XGB. This stability in performance demonstrates the robustness of ML-based analyses for AML and MDS, suggesting that even small datasets can be effectively leveraged to explore feature importance and gene expression relationships. In contrast, the results for MPN vs. Control and PCN vs. Control revealed greater variability. For MPN vs. Control, the average accuracy fluctuated significantly across all models, even as the sample size increased, indicating challenges in achieving stable performance due to molecular complexity and group overlap. Similarly, for PCN vs. Control, the average accuracy began to improve around 23 samples, but inconsistencies persisted, particularly for kNN and NBC, limiting confidence in further analyses. While RF and XGB showed relative improvements, the instability across sample sizes underscores the need for cautious interpretation of results for these groups.

## Discussion

This study systematically explored *NF-κB1* gene expression dynamics across various hematological malignancies using statistical methods, dimensionality reduction techniques, ML, and XAI. By integrating these approaches, we identified critical molecular interactions—particularly the *NF-κB1* and *caspase-1* axis—and demonstrated the robustness of ML-based approaches for small datasets, offering new insights into disease-specific regulatory mechanisms.

The initial demographic and laboratory findings revealed distinct clinical and molecular profiles among MPN, AML, MDS, PCN, and control groups. Despite statistically significant differences, notably in *NF-κB1* and *caspase-1* normalized counts, considerable overlaps persisted. These overlaps underscore the limitations of conventional diagnostic methods and visualizations for effectively distinguishing these diseases 40-42, especially in high-dimensional datasets where subtle molecular variations are embedded within complex gene expression networks.

A simple regression analysis performed on a data from a total of 114 BM MNCs showed that *NF-κB1* normalized counts exhibited statistical significance with the following six variables: *BAX, caspase-1, MyD88, NFKB1A, NGAL*, and *TNF* normalized counts. *NF-κB1* normalized counts had the highest level of statistical association with *caspase-1* normalized counts (R2 = 0.390, p <0.0001, Table 4). In sequence, *NGAL* (R2 = 0.165, Table 4) and *BAX* normalized counts (R2 = 0.077, Table 4) exhibited high explanatory power levels. The study also examined the roles of other significant variables in the canonical* NF-κB* pathway 8, 9. MyD88, activated via Toll-like receptor signaling is a key regulator of *NF-κB* signaling 43. Functional polymorphisms of *NFKBIA*, a known inhibitor of *NF-κB*, play an essential role in influencing NF-κB function, demonstrating the association of *NFKBIA* with *NF-κB* in several diseases, such as cancers. 35, 39 Similarly, TNF is instrumental in NF-κB activation, binding to TNF receptors on the cell surface and initiating signaling cascades that lead to NF-κB activation 44.

Correlation analyses revealed that the MPN group consistently exhibited the strongest correlation between *NF-κB1* and *caspase-1* normalized counts, supporting its selection for further dimensionality reduction and ML analyses. By contrast, the weaker correlations in the Control and PCN groups highlighted their heterogeneity and the potential involvement of distinct regulatory mechanisms (Figure 4). To further investigate these molecular dynamics, PCA and t-SNE were applied to AML, MDS, and PCN groups (Figure S1-S3). However, these dimensionality reduction techniques revealed overlapping distributions across all groups. The inability of PCA and t-SNE to achieve clear discrimination reinforced the notion that subtle molecular distinctions may not be readily captured by dimensionality reduction methods alone (Figure 6) 13, 45, 46. Although PCA and t-SNE provided insights into variance-based and local clustering patterns, their inability to achieve clear group separability highlighted their limitations in capturing the complexity of the data.

Given these challenges, the next logical step was to employ ML models to identify more nuanced patterns. Despite initial insights from scatter plots and dimensionality reduction, the data's complexity required more robust analytical tools. ML methods were thus introduced to enhance classification performance and uncover key features that drive group differentiation. Classifiers such as kNN, NBC, RF, and XGB, were employed to uncover higher-order interactions and complex relationships that traditional statistical and dimensionality reduction methods could not discern 47. Coupled with XAI techniques, such as SHAP and permutation feature importances, the ML framework enabled interpretability and the association analysis of biologically meaningful features, including NF-κB and caspase-1 48, 49.

The application of ML methods yielded promising results, as summarized in Table 6. Ensemble models, such as RF and XGB, generally outperformed simpler classifiers like kNN and NBC, especially when distinguishing between disease groups and the control group. Nonetheless, NBC showed remarkable accuracy in AML and MDS classifications, underscoring its suitability for certain small, high-dimensional datasets. The challenges in MPN and PCN classification, as reflected by moderate-to-low performance metrics, echoed the subtle intergroup differences and molecular complexity observed earlier 50, 51. The variability in performance across classifiers further highlighted the importance of selecting robust techniques capable of handling nuanced gene expression profiles.

Permutation analysis consistently identified *NF-κB1* and *caspase-1* normalized counts as key features. While these two features emerged as key regulators, the feature importance patterns differed across groups, emphasizing the intricate nature of gene-disease relationships and suggesting that hybrid or ensemble ML approaches might provide even deeper insights 52.

These findings align with the interpretability results derived from XAI methods. As shown in Figure 9, SHAP analysis pinpointed *caspase-1* as the most influential feature governing *NF-κB1* expression across multiple disease groups. This pattern corroborated the regression results and provided mechanistic insights, highlighting *caspase-1*'s pivotal role in the canonical *NF-κB* pathways across hematological malignancies 8, 9. Importantly, features such as *RAGE*, *NGAL*, and MYD88 emerged with varying influence across different diseases, suggesting that the *NF-κB1* and *caspase-1* interaction occurs within a broader and context-dependent molecular network 53-55. *NF-κB1* gene was reported to be associated with genes such as *NF-κB2*, RELA, RELB, NFKB1A, BTRC, REL, UBC, CUL1, SQSTM1, and CHUK. However, as identified in this study, the relationship between NF-kB1 and caspase-1 would be highlighted in hematologic malignancies such as AML, MDS, MPN, and PCN 56.

The relationship between *NF-κB* and *caspase-1* is complex and multifaceted, with both pathways influencing each other in various ways 57-59. Inflammasomes are innate immune system sensors found in the cytosol of macrophages, granulocytes, dendritic cells and monocytes, are activated by NF-κB, activating caspase-1 59. Conversely, caspase-1 can activate NF-κB signaling through interactions with caspase-7, cleavage of the MyD88-adapter-like protein, or receptor-interacting protein-2 activation 57.

A previous human study reported that NF-κB1 haploinsufficiency is associated with the CVID phenotype and that the levels of NF-κB1 (p50) are tightly regulated 7. Furthermore, while the specific reasons for the phenotypic variability and the variable age of disease onset associated with p50 haploinsufficiency remain unclear, the study suggested the potential existence of modifier genes that could explain these variations 7. Given the results of our study, caspase-1 may regulate the levels of NF-κB1 and serve as one of the modifier genes influencing disease onset related to deficient NF-κB1.

Beyond these molecular insights, the small dataset size inherent in rare disease research introduced analytical challenges. The evaluation of model performance across varying sample sizes, as illustrated in Figure 10, highlighted the overall improvement in classification accuracy with increasing sample sizes across all models (kNN, NBC, RF, and XGBoost). For AML and MDS, the average accuracy across all four models stabilized above 0.8 when the sample size exceeded 15 and 14 for AML and MDS, indicating reliable classification performance. While simpler models such as kNN and NBC exhibited greater variability and sensitivity to small sample sizes, ensemble methods like RF and XGB demonstrated consistent robustness and achieved more stable results across varying sample sizes 60. For PCN, model performance began to improve around 23 samples, though variability persisted, particularly for kNN and NBC. These findings emphasize the importance of ensemble methods in handling small datasets typical of rare disease research and demonstrate the potential for extracting meaningful insights despite the limitations imposed by data scarcity. In contrast, the MPN group continued to show fluctuations in accuracy, underscoring the challenges posed by molecular complexity and group overlap.

Overall, integrating statistical analyses, dimensionality reduction techniques, ML classifiers, and XAI-driven interpretations proved critical for untangling the complex relationships between *NF-κB1* and other cytokine genes. The consistent identification of *NF-κB1* and *caspase-1* normalized counts as central players across multiple analytical methods—regression, correlation, ML classification, and SHAP interpretations—highlights their pivotal role in inflammation and apoptosis pathways. This universality across disease groups underscores their potential as key targets for therapeutic intervention in hematological malignancies.

Importantly, this study demonstrates that meaningful and stable analyses can be achieved even with small datasets, addressing a fundamental challenge in rare disease research. The systematic evaluation of model performance across varying sample sizes revealed that ensemble methods, particularly RF and XGB, can achieve robust and stable classification accuracy with as few as 15-24 samples for AML and MDS. This underscores the feasibility of deriving biologically meaningful insights even under data-limited scenarios, a critical consideration in rare disease research. By demonstrating the utility of ML-based approaches for identifying key molecular interactions, particularly the NF-κB-caspase-1 axis, this study provides a strong foundation for future applications in precision medicine. Importantly, these results reinforce the viability of leveraging ensemble models to overcome the challenges posed by small datasets, offering a reliable strategy for refining diagnostic tools and guiding targeted therapeutic interventions in resource-limited clinical settings. By focusing on the *NF-κB* and *caspase-1* axis, this work deepens our understanding of disease-specific regulatory mechanisms and highlights their therapeutic potential. Importantly, it demonstrates that ML-based approaches can extract reliable insights from limited data, offering a feasible strategy for precision medicine applications in rare hematological malignancies. These findings provide a strong foundation for future studies aimed at refining diagnostic tools and developing innovative therapeutic hypotheses, even in resource-constrained environments.

## Conclusion

This study demonstrates the efficacy of integrating traditional statistical methods, ML, and XAI to unravel the complex gene interactions in hematological malignancies, with a specific focus on the *NF-κB1* and *caspase-1* axis. By identifying *caspase-1* normalized counts as the most influential factor governing *NF-κB1* gene activity across disease groups, our findings highlight their central role in inflammation and apoptosis pathways. Group-specific regulatory mechanisms, uncovered through SHAP and feature importance analyses, provide additional insights into disease-specific molecular signatures.

Addressing the challenges of small sample sizes inherent in rare disease research, this study establishes a minimum threshold of 15 samples for robust classification, particularly in AML and MDS. These results underscore the potential of ensemble ML models like RF and XGB to achieve stable and interpretable outcomes even in data-limited environments. Further validation in similar sized and independent cohorts will be essential to translate these findings into practical clinical applications.

## Supplementary Material

Supplementary figures.

## Figures and Tables

**Figure 1:**
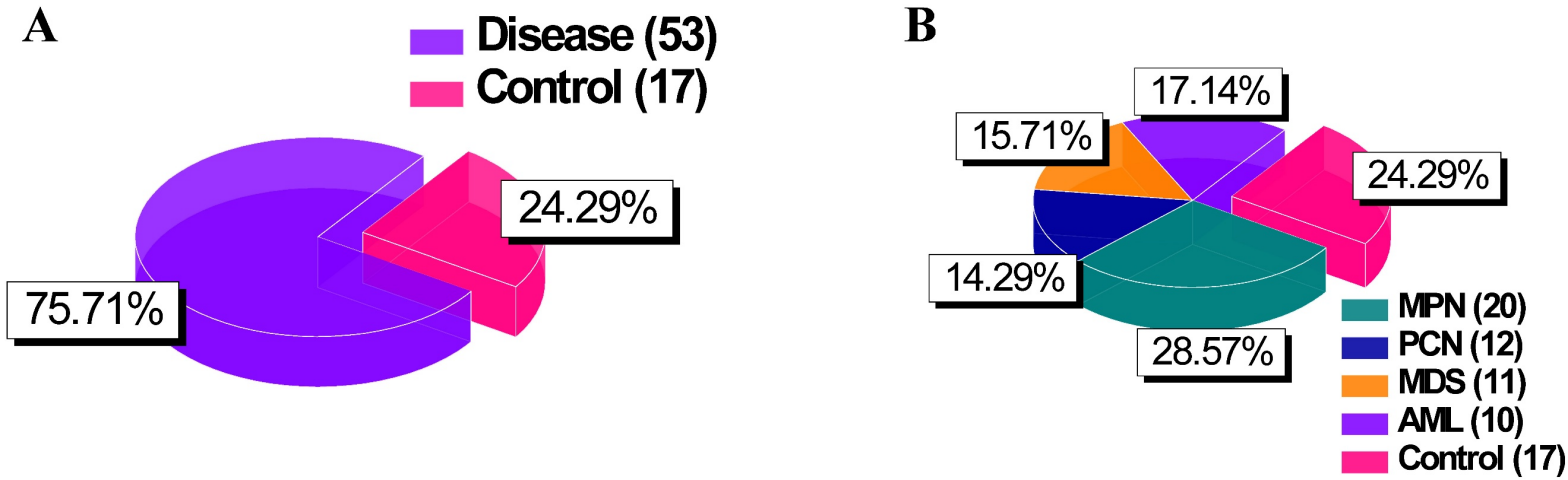
Pie chart of group distributions. (A) Comparison between “Disease” (combining groups 1 to 4) and “Control” groups. (B) Detailed breakdown of the “Disease” category into its constituent groups. Abbreviations: MPN, myeloproliferative neoplasm; PCN, plasma cell neoplasm; MDS, myelodysplastic syndrome; AM, acute myeloid leukemia.

**Table 1:** Patient demographic features and laboratory parameters.

Characteristics	Value
Age (y)	63 (47, 74)
Men/Women	67/47
Hb (g/L)	106.5 (86, 130.3)
WBC count (109/L)	5.65 (3.14, 10.59)
Neutrophil counts (109/L)	2.70 (1.39, 5.92)
Platelet count (109/L)	179.0 (81.8, 322.5)
BM cellularity	45 (25, 80)
M:E ratio	2.4 (1.5, 4.0)
*NF-κB1* gene normalized counts	87.78 (57.06, 172.50)
Disease entities (n = 114)	MPN† (n = 20)
	AML (n = 12)
	MDS (n = 11)
	PCN (n = 10)
	Control‡ (n = 17)
	Others* (n = 44)

Quantitative data are represented as the median (quartile1 [Q1], Q3) values; ^†^MPN included CML (n = 8), PV (n = 4), ET (n = 4), PMF (n = 3), and MPN-U (n = 1); ^‡^The control group comprises patients with lymphoma without BM involvement (n = 16) or a normocellular marrow without hematological malignancy according to the BM smear and pathological review (n = 1); ^*^Others include the involvement of lymphoma in bone marrow (n = 10), hypocellular marrow (n = 8), hypercellular marrow (n = 3), aplastic anemia (n = 3), chronic lymphocytic leukemia (n = 3), immune thrombocytopenia (n = 2), megakaryocytic hyperplasia (n = 2), histiocytosis with some hemophagocytosis (n = 2), B-lymphoblastic leukemia (n = 1), hypereosinophilic syndrome (n = 1), idiopathic hypereosinophilia (n = 1), idiopathic cytopenia of undetermined significance (n = 1), and decreased megakaryopoiesis (n = 1). **Abbreviations**: AML, acute myeloid leukemia; BM, bone marrow; CML, chronic myeloid leukemia; ET, essential thrombocythemia; Hb, hemoglobin; MDS, myelodysplastic syndrome; MPN, myeloproliferative neoplasm; MPN-U, MPN-unclassifiable; PCN, plasma cell neoplasm; PMF, primary myelofibrosis; PV, polycythemia vera; WBC, white blood cell

**Table 2:** Patient demographic features and laboratory parameters of each hematological malignancy and control group (n = 70).

	MPN† (n = 20)	AML (n = 12)	MDS (n = 11)	PCN (n = 10)	Control‡ (n = 17)	*p*-value*
Sex	10 men	11 men	11 men	10 men	13 men	0.030
	10 women	1 woman	0 women	0 women	4 women	
Age (y)	55 (39, 63)	59 (42, 75)	70 (56, 81)	72 (59, 78)	65 (47, 73)	0.068
Hb (g/L)	124 (104, 145)	79 (66, 85)	86 (68, 88)	97 (87, 114)	123 (105, 134)	< 0.0001
WBC count (109/L)	19.31 (10.34, 109.34)	3.37 (2.25, 17.86)	2.38 (1.37, 3.29)	5.04 (4.45, 6.73)	6.87 (4.30, 9.07)	< 0.0001
Neutrophil count (109/L)	14.43 (7.41, 70.51)	0.74 (0.22, 1.76)	0.77 (0.49, 1.09)	3.10 (1.98, 4.59)	3.88 (2.48, 6.39)	< 0.0001
Platelet count (109/L)	620 (403, 876)	49 (24, 131)	78 (31, 138)	179 (112, 198)	288 (227, 337)	< 0.0001
CRP (mg/dL)	0.100 (0.030, 0.743)	8.390 (1.086, 13.385)	0.460 (0.258, 1.330)	0.180 (0.105, 1.180)	0.180 (0.070, 2.095)	0.003
eGFR|| (mL/min/1.73m2)	94.50 (79.67, 111.50)	86.72 (68.18, 95.42)	80.95 (54.03, 104.88)	61.26 (30.35, 81.21)	93.09 (84.54, 105.51)	0.001
*NF-κB1* gene normalized counts	54.25 (39.21, 79.56)	177.65 (55.37, 231.33)	167.54 (113.46, 288.56)	133.49 (78.77, 270.60)	56.75 (43.55, 76.98)	< 0.0001
*Caspase-1* gene normalized counts	162.55 (87.64, 403.05)	451.77 (158.09, 766.34)	805.92 (339.01, 1144.15)	453.48 (258.82, 952.57)	199.82 (120.76, 213.35)	< 0.0001
*BAX* gene normalized counts	463.45 (253.51, 931.39)	931.85 (580.05, 1609.36)	639.08 (309.94, 885.57)	546.53 (317.65, 1672.87)	396.47 (292.88, 559.31)	0.021
*NGAL* gene normalized counts	34490.76 (23170.44, 48587.74)	324.80 (114.39, 5278.39)	5037.18 (4522.28, 7581.07)	17372.95 (2745.81, 44363.49)	45859.73 (31338.67, 55617.08)	< 0.0001

Quantitative data are represented as the median (quartile1 [Q1], Q3) values; ^†^MPN includes CML (n = 8), PV (n = 4), ET (n = 4), PMF (n = 3), and MPN-U (n = 1); ^‡^The control group comprises patients with lymphoma without BM involvement (n = 16) or normocellular marrow without hematological malignancy according to the BM smear and pathological review (n = 1);^ *^ for gender, the Pearson Chi-squared test was used, for age, Hb, platelet count, and the eGFR variable, a one-way ANOVA was performed, and for the other variables, the Kruskal-Wallis H-test was performed; ^||^, eGFR was calculated using the CKD-EPI equation. **Abbreviations**: AML, acute myeloid leukemia; BAX, BCL2-associated X; BM, bone marrow; CKD-EPI, Chronic Kidney Disease Epidemiology Collaboration equation; CML, chronic myeloid leukemia; CRP, C-reactive protein; eGFR, estimated glomerular filtration rate; ET, essential thrombocythemia; Hb, hemoglobin; MDS, myelodysplastic syndrome; MPN, myeloproliferative neoplasm; MPN-U, MPN-unclassifiable; NF-κB1, nuclear factor kappa light chain enhancer of activated B cells 1; NGAL, neutrophil gelatinase-associated lipocalin; PCN, plasma cell neoplasm; PMF, primary myelofibrosis; PV, polycythemia vera; WBC, white blood cell

**Table 3:** Pairwise comparison of six numerical variables in five groups: MPN, AML, MDS, PCN, and control

Variable	Comparison groups	p-value*
Hb	MPN vs. AML	<0.0001
	MPN vs. MDS	<0.0001
	MPN vs. PCN	0.027
	AML vs. Control	<0.0001
	MDS vs. Control	0.001
WBC count	MPN vs. AML	0.003
	MPN vs. MDS	<0.0001
	MDS vs. Control	0.009
Neutrophil count	MPN vs. AML	<0.0001
	MPN vs. MDS	<0.0001
	AML vs. Control	0.003
	MDS vs. Control	0.006
Platelet count	MPN vs. AML	<0.0001
	MPN vs. MDS	<0.0001
	MPN vs. PCN	<0.0001
	MPN vs. Control	<0.0001
CRP	MPN vs. AML	0.001
eGFR	MPN vs. PCN	0.002
	PCN vs. Control	0.001

^*^p-value was modified by Bonferroni correction. **Abbreviations**: AML, acute myeloid leukemia; CRP, C-reactive protein; eGFR, estimated glomerular filtration rate; Hb, hemoglobin; MDS, myelodysplastic syndrome; MPN, myeloproliferative neoplasm; NGAL, neutrophil gelatinase-associated lipocalin; PCN, plasma cell neoplasm; WBC, white blood cell

**Table 4:** Simple regression analysis of normalized counts of *NF-κB1* and ten cytokine genes in 114 bone marrow mononuclear cells

Gene Name	R^2^	*P*-value^†^	Normalized counts^‡^
*BAX*	0.077	0.003^*^	559.31 (345.47, 880.01)
*BCL2L1*	0.030	0.066	1564.99 (1048.08, 3438.39)
*Caspase-1*	0.390	<0.0001^*^	344.35 (180.33, 629.68)
*IFNB1*	0.003	0.574	6.06 (2.89, 15.68)
*MyD88*	0.188	<0.0001^*^	1172.02 (794.85, 1689.91)
*NFKBIA*	0.114	<0.0001^*^	3508.07 (2126.11, 5518.07)
*NGAL*	0.165	<0.0001^*^	29320.74 (7367.25, 46058.72)
*NLRP3*	0.004	0.514	520.03 (325.66, 861.76)
*RAGE*	0.017	0.161	29.30 (16.20, 57.11)
*TNF*	0.070	0.005^*^	204.65 (121.95, 339.48)

^†^ Statistically significant (*P <*0.05); ^‡^Normalized counts are represented as the median (quartile1 [Q1], Q3 values); ^*^ these cytokine genes were included as independent factors in multiple regression models; *P*-values <0.05 were typed in boldface. **Abbreviations**: *BAX*, bcl-2-associated X protein; *BCL2L1*, bcl-2-like 1; *IFNB1,* interferon beta 1; *NGAL*, neutrophil gelatinase-associated lipocalin; Q, quartile; *RAGE*, receptor for advanced glycation end products

**Table 5:** Regression analysis models of the relationship between the normalized counts of *NF-κB1* and those of cytokine genes in the bone marrow.

	Coefficient	*t*-value	*P*-value^†^	VIF	Adj *R*^2^	AIC
Constant	75.034	2.972	0.004		0.434	1068.667
*BAX* normalized counts	0.030	2.378	0.019	1.032		
*Caspase-1* normalized counts	0.157	6.843	< 0.0001	1.196		
*NGAL* normalized counts	-0.001	-2.271	0.025	1.189		

^†^Statistically significant (*P <*0.05). **Abbreviations**: Adj, adjusted; AIC, Akaike's information criterion; VIF, variance influence factor

**Table 6:** Performance metrics for k-Nearest Neighbors (kNN), Naïve Bayes Classifier (NBC), Random Forest (RF), and XGBoost (XGB) applied to distinguish between the control group and each hematological malignancy group (MPN, AML, MDS, PCN). Metrics include accuracy, precision, recall, F1 score, and area under the ROC curve (AUC), summarizing the classification performance of each model across different comparisons.

Group Pair	Model	Accuracy	Precision	Recall	F1 Score	ROC AUC
MPN vs Control	kNN	0.48	0.50	0.48	0.46	0.60
	NBC	0.60	0.66	0.58	0.59	0.66
	RandomForest	0.62	0.68	0.75	0.67	0.81
	XGB	0.59	0.65	0.79	0.67	0.67
AML vs Control	kNN	0.90	1.00	0.82	0.88	0.95
	NBC	1.00	1.00	1.00	1.00	1.00
	RandomForest	1.00	1.00	1.00	1.00	1.00
	XGB	0.93	0.93	0.90	0.89	0.93
MDS vs Control	kNN	0.78	0.73	0.58	0.61	0.91
	NBC	0.89	0.87	0.96	0.90	0.97
	RandomForest	0.87	0.70	0.70	0.67	1.00
	XGB	0.86	0.73	0.66	0.67	0.84
PCN vs Control	kNN	0.62	0.40	0.30	0.33	0.70
	NBC	0.71	0.75	0.78	0.66	0.76
	RandomForest	0.71	0.67	0.78	0.65	0.91
	XGB	0.67	0.65	0.78	0.63	0.94

Abbreviations: MPN, myeloproliferative neoplasm; AML, acute myeloid leukemia; MDS, myelodysplastic syndrome; PCN, plasma cell neoplasm; kNN, k-nearest neighbors; NBC, naïve Bayes classifier; XGB, extreme gradient boosting.

## Abbreviations

AML: acute myeloid leukemia
BAX: bcl-2-associated X protein
BCL2L1: bcl-2-like 1
BM: bone marrow
CML: chronic myeloid leukemia
CRP: C-reactive protein
eGFR: estimated glomerular filtration rate
ET: essential thrombocythemia
Hb: hemoglobin
IFNB1: interferon beta 1
MDS: myelodysplastic syndrome
ML: machine learning
MPN: myeloproliferative neoplasm
MPN-U: MPN-unclassifiable
NF-κB: nuclear factor kappa light chain enhancer of activated B cells
NGAL: neutrophil gelatinase-associated lipocalin
PCN: plasma cell neoplasm
PMF: primary myelofibrosis
PV: polycythemia vera
Q: quartile
RAGE: receptor for advanced glycation end products
VIF: variance influence factor
WBC: white blood cell
