# Supplementary material for: Analysis of the Relationship Between NF-κB1 and Cytokine Gene Expression in Hematological Malignancy: Leveraging Explained Artificial Intelligence and Machine Learning for Small Dataset Insights
Source: Int J Med Sci. 2025 Apr 13;22(9):2208–26. doi: 10.7150/ijms.109493 (PMC12035828; doi:10.7150/ijms.109493)
Supplement: Supplementary file 1 — Supplementary figures. [file ijmsv22p2208s1.pdf]

Supplementary figures:

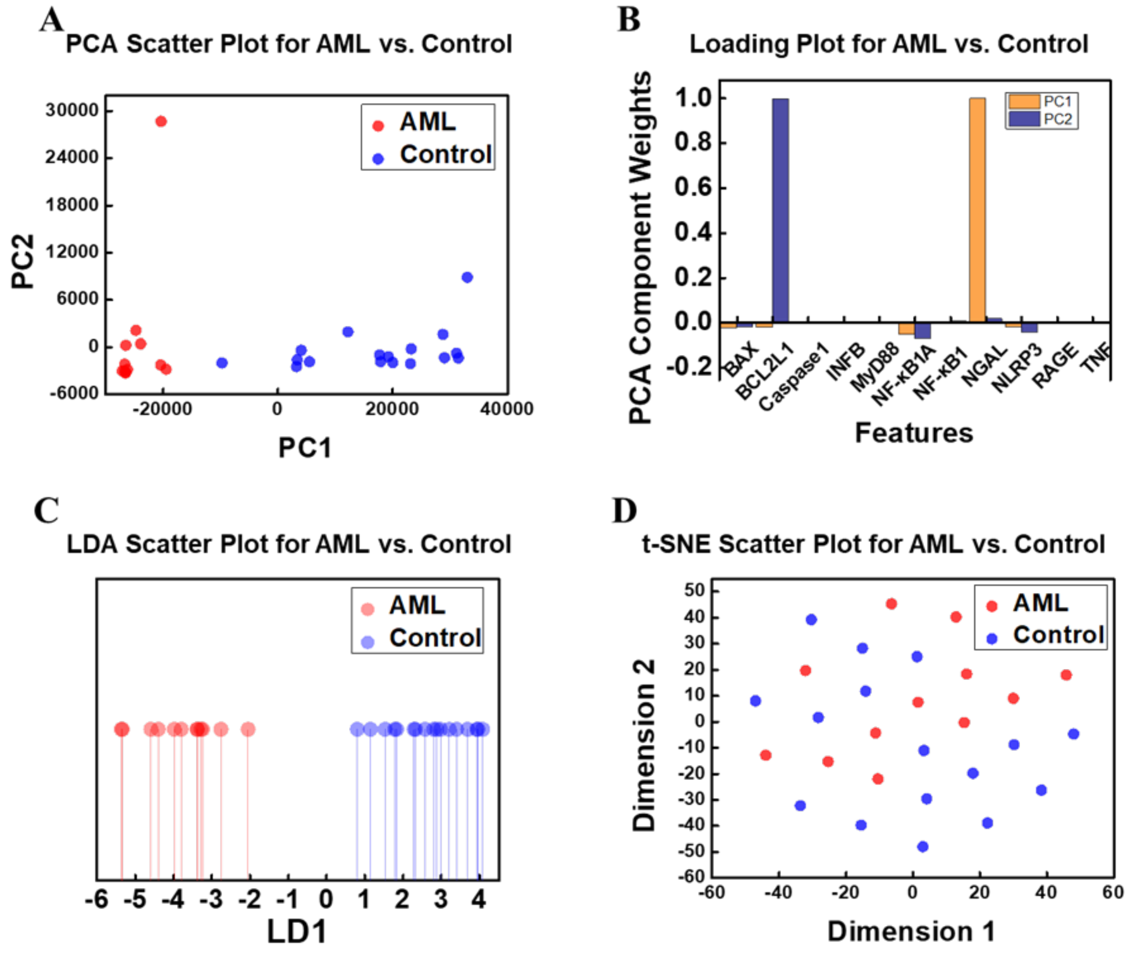

Figure S1 PCA, Loading Plot, LDA, and t-SNE analysis for AML vs Control group. (A) PCA scatter plot highlighting separation trends between AML (red) and control (blue) samples. (B) Loading plot showing key gene contributions (e.g., Caspase-1 and NF-κB1) to the principal components. (C) LDA scatter plot demonstrating improved linear separation of AML and control samples. (D) t-SNE visualization displaying AML-specific clustering with minor overlap.

Abbreviations: AML, acute myeloid leukemia;PCA, principal component analysis; PC, principal component; LDA, linear discriminant analysis; t-SNE, t-distributed stochastic neighbor embedding; LD, linear discriminant

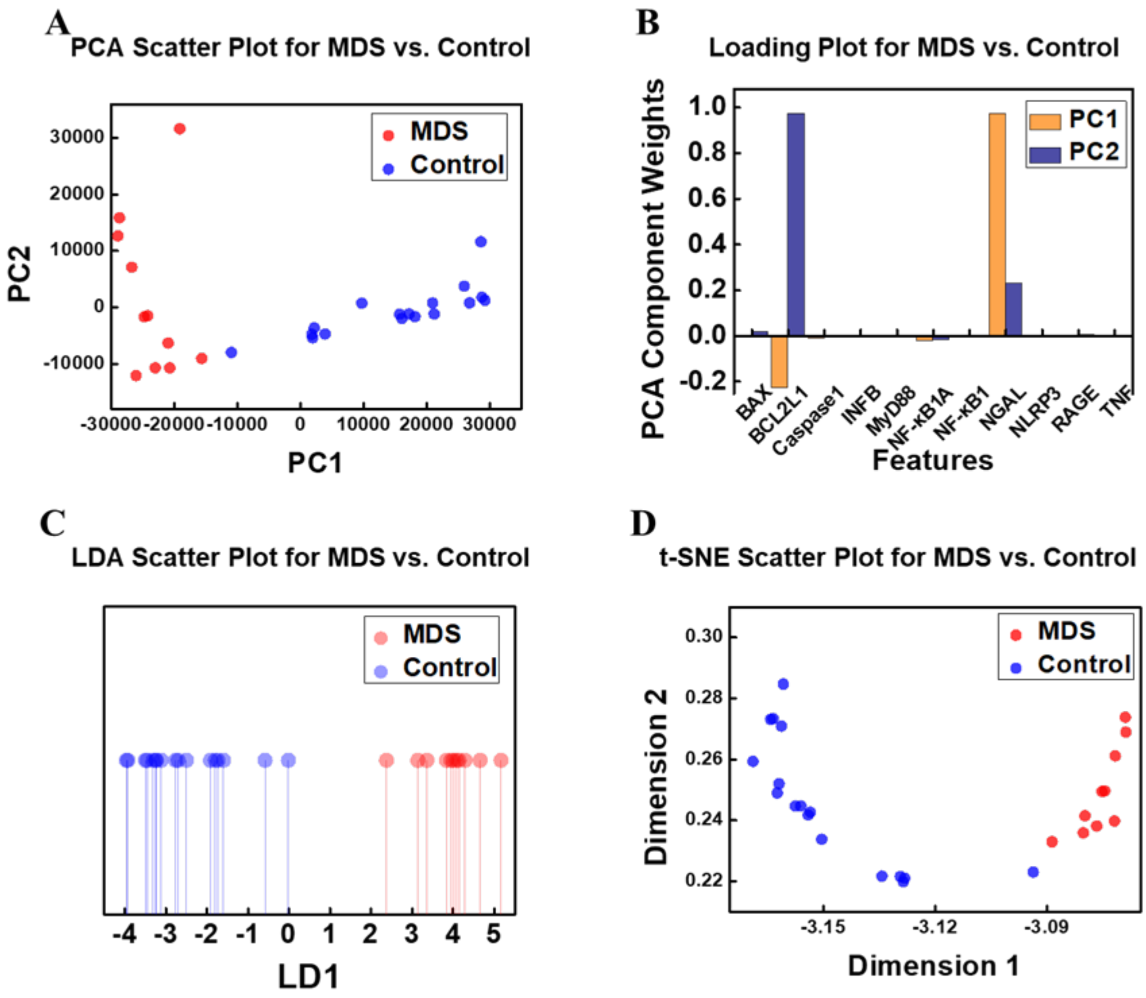

Figure S2 PCA, Loading Plot, LDA, and t-SNE analysis for MDS vs Control group. (A) PCA scatter plot illustrating clustering of MDS (red) and control (blue) samples. (B) Loading plot identifying Caspase-1 and RAGE as primary contributors to PC1. (C) LDA scatter plot showing enhanced linear separation between MDS and control samples. (D) t-SNE visualization reflecting MDS clustering with some non-linear overlaps.

Abbreviations: PCA, principal component analysis; PC, principal component; LDA, linear discriminant analysis; t-SNE, t-distributed stochastic neighbor embedding; LD, linear discriminant

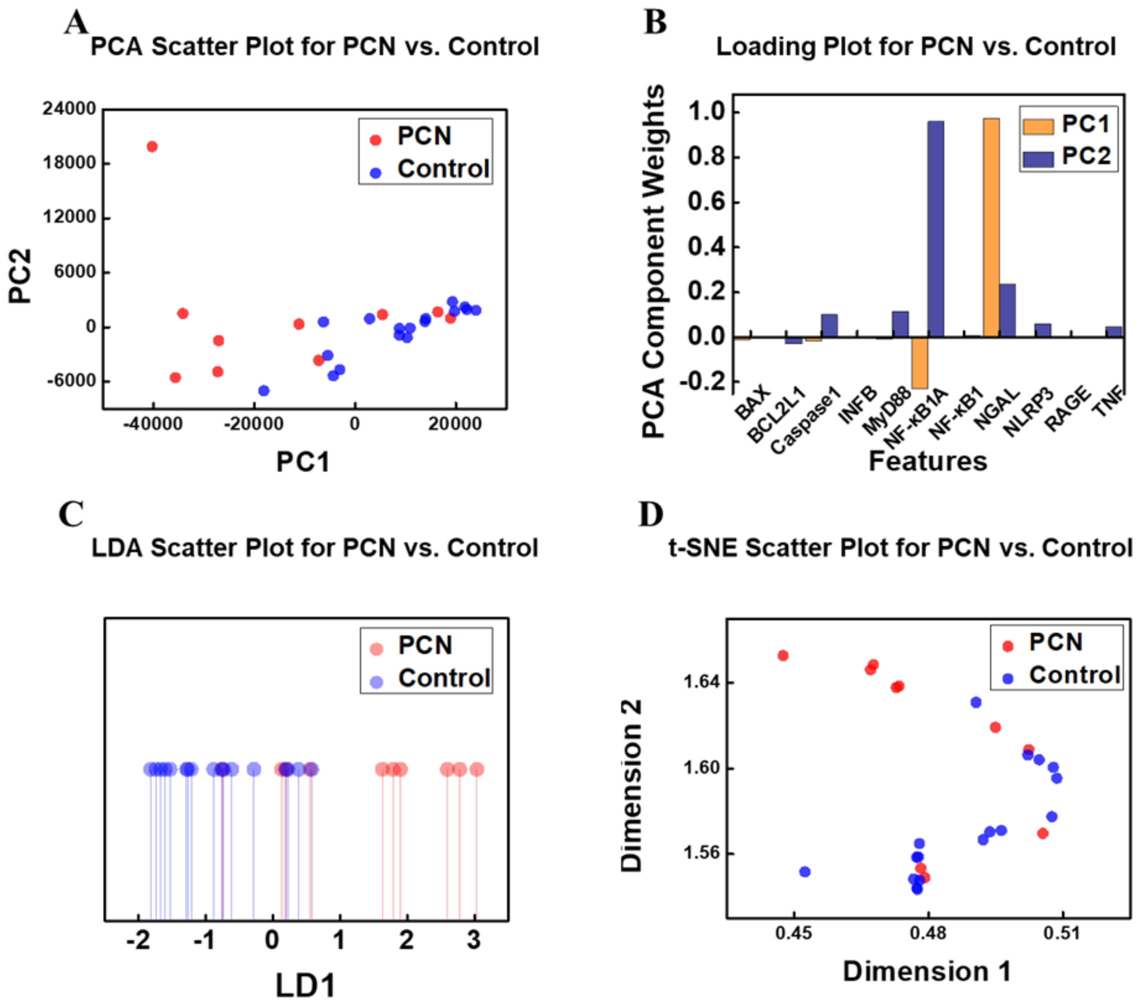

Figure S3 PCA, Loading Plot, LDA, and t-SNE analysis for PCN vs Control group. (A) PCA scatter plot showing significant overlap between PCN (red) and control (blue) samples. (B) Loading plot emphasizing Caspase-1 and NF-κB1 contributions to PC1. (C) LDA scatter plot displaying moderate separation between PCN and control samples. (D) t-SNE visualization highlighting PCN clustering with substantial intergroup overlap.

Abbreviations: PCN, plasma cell neoplasm; PCA, principal component analysis; PC, principal component; LDA, linear discriminant analysis; t-SNE, t-distributed stochastic neighbor embedding; LD, linear discriminant
